# Heat-stress-induced ROS in maize silks cause late pollen tube growth arrest and sterility

**DOI:** 10.1016/j.isci.2024.110081

**Published:** 2024-05-22

**Authors:** Wen Gong, Mhaned Oubounyt, Jan Baumbach, Thomas Dresselhaus

**Affiliations:** 1 Cell Biology and Plant Biochemistry, University of Regensburg, 93040 Regensburg, Germany; 2 Faculty of Mathematics, Informatics and Natural Sciences, University of Hamburg, 22607 Hamburg, Germany

**Keywords:** Biological sciences, Plant biology, Plant development, Plant physiology

## Abstract

The reproductive phase of plants is highly sensitive to ambient temperature stresses. To investigate sensitivity of female reproductive organs in grass crops during the pollination phase, we exposed the elongated stigma (silk) of maize to ambient environment at the silking stage. Moderate heat stress causes cell death of silk hair cells but did not affect early pollen tube growth inside the silk. Late pollen tube growth arrest was observed, leading to sterility. Heat stress causes elevated levels of reactive oxygen species (ROS) in silks, whose levels can be reduced by scavengers partly restoring pollen tube growth and fertility. A number of biological processes including hydrogen peroxide catabolic processes and bHLH transcription factor genes are downregulated by heat stress, while some NAC transcription factor genes are strongly upregulated. In conclusion, this study now provides a basis to select genes for engineering heat-stress-tolerant grass crops during the pollination phase.

## Highlights

• Late pollen tube growth in maize silks exposed to heat stress leads to sterility
• Heat stress causes elevated ROS levels in maize silks correlated with sterility
• Fertility can be partly restored by spraying ROS scavengers
• Peroxidases, bHLH, and NAC transcription factors are misregulated after heat stress

## Graphical abstract

**Figure:**
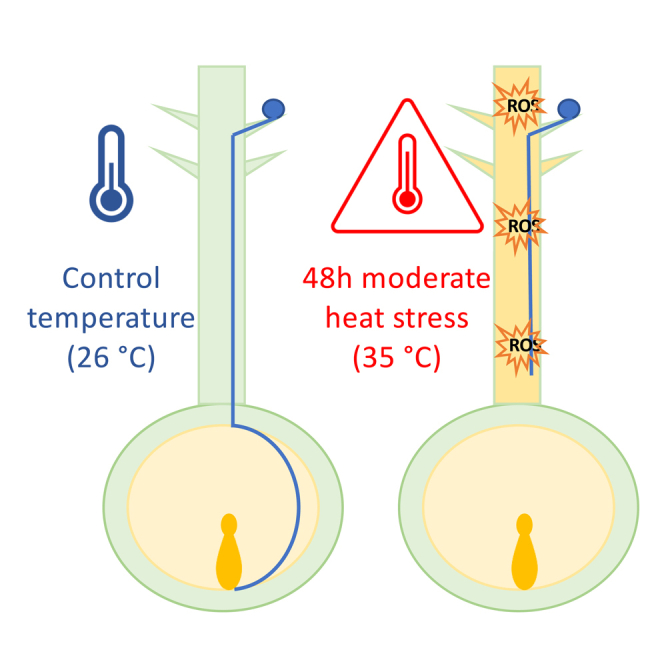

## Introduction

Depending on the plant species and ecotype, a rise in temperature above a critical threshold level for a period of time results in irreversible damage to plant growth, development, and reproduction.^1^^,^^2^ High temperature significantly decreases the yield of cereal crops such as maize (*Zea mays*) and due to global climate change is predicted to occur more frequently in the future.^3^^,^^4^^,^^5^^,^^6^^,^^7^ Studies indicated that heat waves will become more frequent, more intense, and longer lasting.^8^^,^^9^^,^^10^ As a consequence, a significant reduction in crop yield will be caused by heat stress if we do not manage to generate heat-stress-tolerant crop plants.^11^^,^^12^

The yield of agricultural crops such as maize, wheat (*Triticum* spec.), barley (*Hordeum vulgare*), rice (*Oryza sativa*), soybean (*Glycine max*), oil seed rape (*Brassica napus*), and many others mainly relies on their production of seeds. Seed set relies on the production of functional flowers containing male and female gametophytes (pollen and embryo sacs, respectively), successful fertilization mechanisms, and subsequent growth and development of both fertilization products, embryo and endosperm, respectively. The reproductive phase including meiosis, pollen development, their germination, and tube growth as well as fertilization is particularly sensitive to temperature fluctuations.^13^^,^^14^^,^^15^^,^^16^ A number of studies have been performed already to understand how heat stress affects reproduction in maize and other plants. It was shown that especially pollen development and pollination are susceptible to heat stress,^16^^,^^17^^,^^18^^,^^19^^,^^20^^,^^21^^,^^22^^,^^23^ and pollen was even suggested to be the most sensitive tissue/cell affected by heat stress.^24^ After exposure to heat stress, pollen starch content, pollen viability as well as pollen germination and sperm cell transport into tubes were decreased, which causes an increase in the rate of failed pollination.^19^^,^^20^^,^^23^^,^^24^^,^^25^^,^^26^

In contrast to the amount of work carried out on pollen, less research has been done on the development and sensitivity of female reproductive organs under heat stress due to their protection by surrounding tissues and leaves. Maize, as a grass crop model for studying reproduction, is especially suited for such studies.^27^^,^^28^^,^^29^ Its stigmatic tissue (silk) is separated from male flower organs and extends outward from husk leaves at cobs during the silking stage and thus is directly exposed to the ambient environment. Moreover, due to the separation of male and female inflorescences, silks of stressed plants can easily be pollinated, with pollen from unstressed plants allowing to separate stresses occurring during different reproductive stages and fertilization processes. So far, studies have shown that heat stress accelerates the senescence and receptivity of maize silks,^30^^,^^31^ but the underlying molecular mechanisms remain unclear.

The fertilization process in maize begins when compatible pollen grains land on stigmatic papilla hairs (silk hairs). Following pollen grain adhesion, hydration, and germination, pollen tubes penetrate silk hairs and grow through one of the two transmitting tracts toward the female gametophyte.^27^ In maize, pollen tubes grow up to 30 cm within 24 h to reach the ovule, where the double fertilization process occurs.^32^ Pollen tube guidance signals and mechanical structures of female reproductive organs precisely regulate the directed growth and subsequent successful fertilization.^28^^,^^33^^,^^34^

Reactive oxygen species (ROS; e.g., O^2^·−, H_2_O_2_, OH·, ^1^O_2_) serve as signaling molecules at low to modest doses in various biological processes including plant development and reproduction as well as stress responses but cause cell death at high concentrations.^35^^,^^36^^,^^37^ During pollination, ROS derived from both male and female tissues play regulatory roles for communication between the pollen tube and female tissues at various stages, such as pollen hydration and germination at the stigma, pollen tube tip growth in the pistil, pollen tube reception in the female gametophyte, and tube burst during fertilization.^37^^,^^38^^,^^39^^,^^40^^,^^41^^,^^42^^,^^43^^,^^44^^,^^45^^,^^46^ On the other hand, ROS levels are increased in pollen tubes under heat stress conditions, which was shown to inhibit pollen tube growth and pollen tube integrity.^47^ Maintenance of ROS homeostasis is crucial for functional ROS signaling, as intermediate levels of ROS positively regulate signaling, while high levels have cytotoxic effects.^48^ Plants use diverse enzymatic and nonenzymatic antioxidants, which are also called ROS scavengers to prevent toxicity and maintain ROS homeostasis.^47^^,^^48^^,^^49^ Whether ROS signaling crosstalk exists between male and female tissues in maize and which role it might play under heat stress is not known.

This work tested the sensitivity and contribution of female reproductive organs under heat stress in maize. We exposed female reproductive organs of maize at the silking stage to moderate heat stress and studied among others cell viability and death, membrane integrity, pollen germination, and tube growth inside the silk as well as the influence of transient heat stress on seed set. Different ROS species were investigated as well as the influence of ROS scavengers on pollen tube growth. The transcriptome of heat-stressed silks was compared with non-stressed silks to elucidate molecular mechanisms and players regulated by heat stress, ultimately leading to sterility.

## Results

### Heat stress causes cell death and decreases vitality of silk hair cells

**Figure 1:**
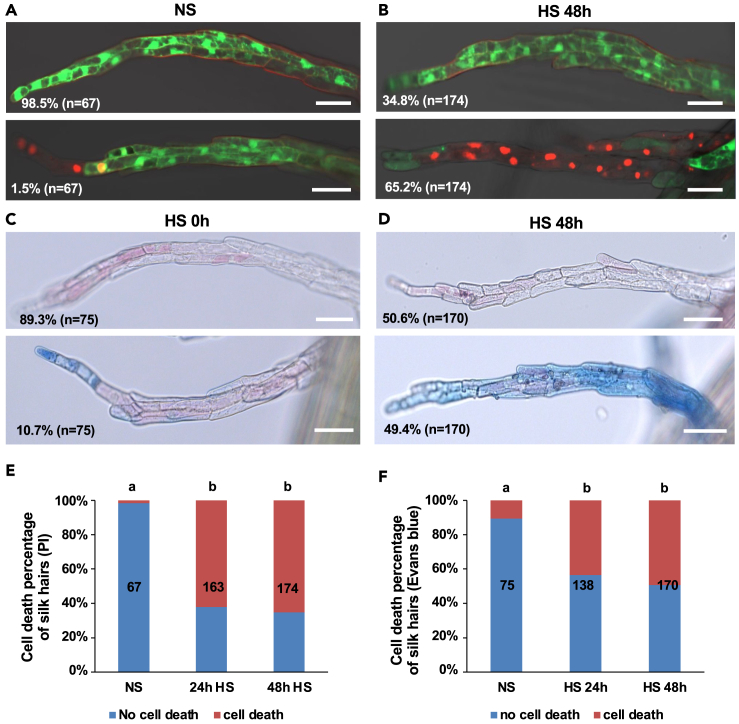
Heat stress causes cell death and decreased cell vitality of maize silk hair cells (A and B) Confocal microscopy of silk hairs stained with fluorescein diacetate (FDA) and counter-stained with propidium iodide (PI). Silks (3 days after silk emergence) before heat stress (NS) (A) and after 48 h heat stress (HS) treatment (B). Upper picture shows a representative silk hair lacking dead cells, whereas the lower picture shows a silk hair with cell death indicated by PI staining in nuclei. Pictures show the merged channels of DIC, FDA, and PI. Scale bars: 50 μm. (C and D) Nomarski microscopy of Evans-blue-stained silk hairs. Silks (3 days after silk emergence) before heat stress (NS) (C) and after 48 h HS treatment (D). Upper picture shows a silk hair lacking dead cells, and lower picture shows a silk hair with cell death indicated by Evans blue staining of cells. Scale bar: 50 μm. (E) Percentage of silk hairs with dead cells (PI-stained nuclei). Number of silk hairs counted are written on each column. Letters on columns indicate significantly associated categories. *p* < 0.01 by the Tukey-Kramer test. (F) Percentage of silk hairs with dead cells (Evans-blue-stained cells). Number of silk hairs counted is written on each column. Letters on columns indicate significantly associated categories. *p* < 0.01 by the Tukey-Kramer test.

To investigate the effect of heat stress on female reproductive organs of maize, we applied moderate heat stress (35°C/25°C day/night rhythm) to plants carrying female flower organs (ears) at 3 days after the emergence of silk. Plants were exposed for 1 or 2 days (24 and 48 h, respectively) to heat stress (HS), whereas plants carrying male flower organs (tassel) were grown at non-stress (control) conditions (26°C/21°C day/night rhythm). To investigate the effect of HS on cell vitality of silk hairs, silks were stained after HS treatment with fluorescein diacetate (FDA) and propidium iodide (PI) solution. Without heat stress (NS), 98.5% (*n* = 67) silk hairs showed viable cells (positive) FDA staining with PI staining in the cell wall; 1.5% silk hairs contained dead cells, which showed PI staining of nuclei and decreased cell vitality according to decreased FDA staining (Figure 1A). The percentage of cell death and lack of cell vitality increased to 62.1% (*n* = 163) after 24 h (h) HS, and further increased to 65.2% (*n* = 174) after 48 h HS (Figures 1B and 1E). HS periods longer than 48 h further increased the cell death rate. Taken together, HS causes increased cell death and decreased cell vitality of silk hair cells.

To further study the effect of HS on silks, we analyzed plasma membrane integrity by Evans blue staining, which can also be used to show cell vitality. At NS conditions, 89.6% (*n* = 66) silk hairs did not show any Evans blue staining inside cells, whereas the other 10.4% silk hairs showed weak staining in a few cells at their tips (Figure 1C). After 24 h HS, 43.5% (*n* = 138) silk hairs showed Evans blue staining in the cytoplasm of cells. The percentage of silk hairs with Evans blue staining inside cells further increased to 49.4% (*n* = 170) after 48 h HS (Figures 1D and 1F).

However, there was no big difference between 24 h and 48 h HS regarding cell death rate (Figure 1F), which is consistent with cell death and vitality rates observed after PI and FDA staining. Taken together, transient HS for only 1 to 2 days causes decreased plasma membrane integrity and cell vitality of silk hair cells, while up to 50% cells appear damaged or dead.

To confirm that the increased cell death rate is indeed caused by HS and is not related to a process of developmental programmed cell death (dPCD) during the 48-h treatment^50^ that can be observed from 7 days after silk emergence,^51^ plants containing silks 3 days after emergence were used and kept at control conditions for another 48 h and stained with FDA and PI; 98.8% and 98.5% silk hairs, respectively, contained fully vital cells and lacked any signs of cell death (Figures S1A–S1D). This is similar to the time point before the treatment, indicating that dPCD did not play a role, and HS is indeed the cause for increased cell death and decreased cell vitality. To investigate whether HS-induced cell death is linked to PCD, terminal deoxynucleotidyl transferase (TdT) dUTP nick end labeling (TUNEL) assay was carried out to show the occurrence of double-strand DNA breaks. Before HS treatment, 7.9% (*n* = 63) silk hairs contained cells with DNA fragmentation (Figure S2C). After 48 h HS treatment, the percentage of silk hairs with DNA fragmentation only slightly increased to 9.9% (*n* = 81) (Figure S2D). This finding suggests that the type of cell death caused by HS is different from dPCD.

### Heat stress on silks causes sterility due to late pollen tube growth arrest

**Figure 2:**
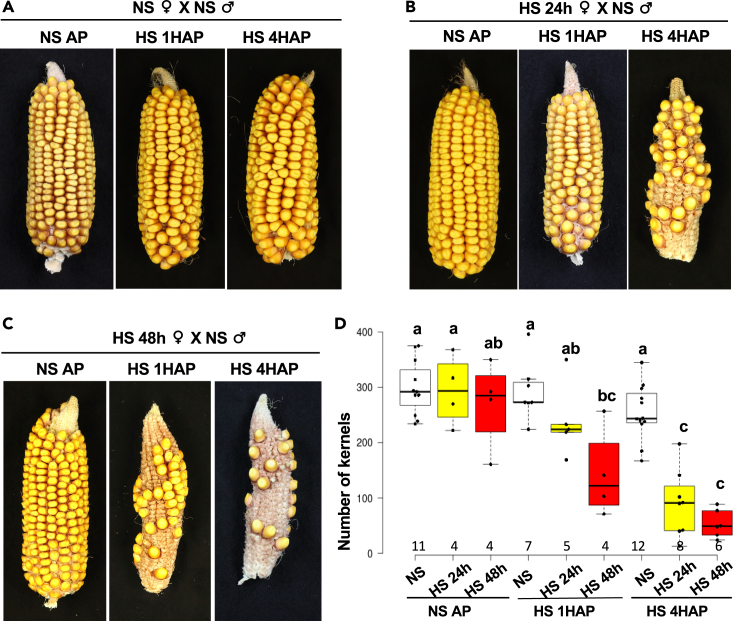
Heat stress on silks causes seed set reduction in maize (A–C) Cobs harvested about 1 month after indicated crossings. Representative examples of cobs are shown from crossings of silks (♀) from non-stress condition (NS) (A), or after 24 h heat stress (HS 24 h) (B), or after 48 h heat stress (HS 48 h) (C) with NS pollen (♂). Plants were exposed for another 1 h or 4 h HS after pollination (HS 1HAP or HS 4HAP), respectively, or directly transferred to control conditions after pollination (NS AP). (D) Quantification of kernel numbers from indicated crosses. Data are presented as mean ± SD. Letters above columns indicate different statistical groups with *p* < 0.05 as analyzed by two-way ANOVA and the Tukey-Kramer test. Numbers under columns indicate the numbers of cobs quantified.

Seed set is the most important criterion to measure fertility, which determines and limits the yield of crop plants. Therefore, we next analyzed seed set of maize cobs after HS exposure. Full seed set was obtained in cobs from NS silks crossed with non-stressed pollen, regardless whether silks were exposed after pollination for 1 h and 4 h to HS, respectively (Figure 2A). However, seed set was dramatically reduced when silks were exposed for 24 h to HS before pollination and another 4 h HS after pollination compared to the removal of exposure (0 h) or only another 1 h HS after pollination (Figure 2B). Notably, seed set was still full in cobs when silks were exposed to 48 h HS before pollination, but when pollen tube growth occurred at control conditions, seed set was reduced to about one-fourth after an additional 1h HS after pollination and even further reduced after additional 4 h HS exposure (Figures 2C and 2D). This indicates that moderate HS on silks for short periods during the pollen germination and growth phase causes a strong reduction in seed setting and thus sterility. Sterility is increased during longer HS periods both, before, and after pollination.

**Figure 3:**
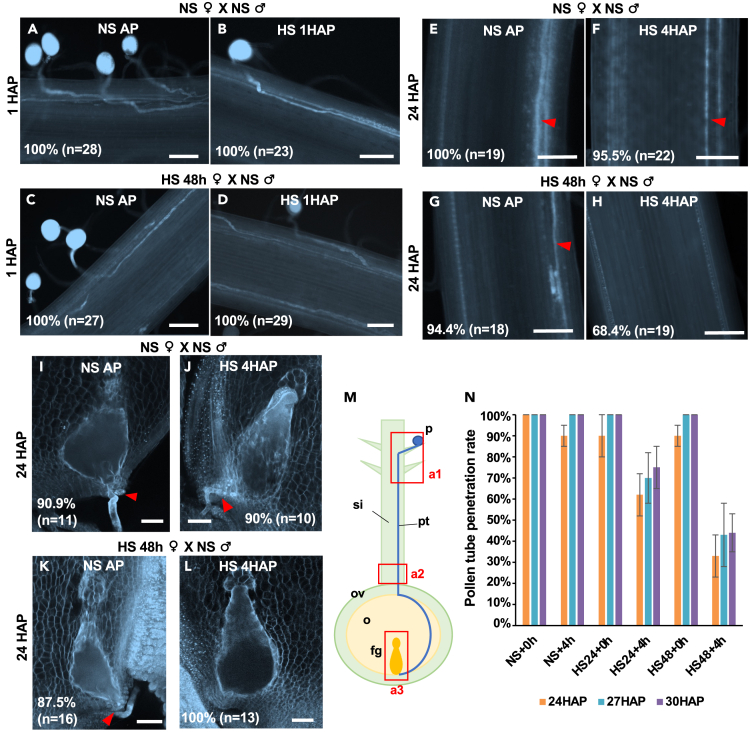
Heat stress on maize silks inhibits late pollen tube growth (A–D) Fluorescent microscopy of aniline-blue-stained silks 1 h after pollination. (A) Non-stressed (NS) silks (3 days after emergence) were pollinated with NS pollen, then kept in NS condition after pollination (AP); (B) NS silks were pollinated with NS pollen, then treated with heat stress 1 h after pollination (HS 1h AP); (C–D) silks (3 days after emergence) after 48 h heat stress treatment (HS 48 h) were pollinated with NS pollen, then kept in NS condition after pollination for 1 h (C) or treated with HS 1 h after pollination (D). Scale bars: 100 μm. (E–H) Fluorescent microscopy of aniline-blue-stained silks at the 1 to 2 cm proximal region (abscission zone) at 24 h after pollination (HAP). Silks (3 days after emergence) at NS conditions before and after pollination (E) or treated with 4 h HS after pollination (HS 4 h AP) (F); silks (3 days after emergence) after 48 h HS (HS 48 h) were pollinated, then kept in NS conditions after pollination (NS AP) (G), or at 4 h HS after pollination (H). Red arrowhead indicates pollen tubes in the transmitting tract of silks. Scale bars: 100 μm. (I–L) Fluorescent microscopy of aniline blue staining of ovule section, which contains the female gametophyte and the micropylar region at 24 HAP. Silks (3 days after emergence) at NS conditions before and after pollination (I) or treated with 4 h HS after pollination (HS 4 h AP) (J); silks (3 days after emergence) after 48 h HS (HS 48 h) were pollinated, then kept at NS conditions after pollination (NS AP) (K), or 4 h HS after pollination (L). Red arrowheads indicate pollen tubes at the micropylar region. Scale bars: 50 μm. (M) Schematic drawing of pollen tube growth in maize silks toward the ovule. Red squares indicate observed areas for aniline blue staining: a1 (A–D), a2 (E–H), a3 (I–L). Abbreviations: fg: female gametophyte; o: ovule; ov: ovary; p: pollen; pt: pollen tube; si: silk. (N) Histogram showing percentage of pollen tube penetration at the proximal region (a2) of silks at 24, 27, and 30 HAP. NS/HS indicate silks under non-stress or heat stress condition. +0 h/4 h indicate 0 h or 4 h heat stress after pollination. Data are presented as mean ± SD.

Pollen tube germination, growth, and guidance in the transmitting tract of the silk is a multi-stage process including complex interaction between the male gametophyte (pollen tube) as well as sporophytic tissue and female gametophyte.^52^ Therefore, we next analyzed the effect of HS on pollen germination and tube growth to elucidate the cause of sterility. Aniline blue staining was used to determine the penetrance of pollen tubes into the silk and further growth toward the transmitting tract. At control conditions, NS silks were pollinated with NS pollen and pollen tube growth into the transmitting tract observed for 1 h after pollination (1 HAP) either at NS and HS conditions. At both conditions, multiple pollen germinated, and tubes were guided into the transmitting tract (Figures 3A and 3B; region a1 in Figure 3M). Pollen tube growth inside the transmitting tract was observed in silks previously exposed for 24 h and 48 h to HS, respectively, and observed either directly or after another 1 h HS exposure after pollination (Figures 3C, 3D, S3A, and S3B). Again, multiple pollen tubes penetrated the transmitting tract with a slightly lower number compared to NS conditions. This suggests that moderate heat stress has no significant effect on the early phase of pollen germination at the silk hair surface, for tube penetration as well as guidance into the transmitting tract.

After arrival in the transmitting tract, pollen tubes of grasses continue to grow through the transmitting tract until they reach the ovule and are further guided to the micropylar region of the female gametophyte^52^ (Figure 3I). Therefore, we next investigated the effect of HS on late growth of pollen tubes in the transmitting tract before pollen tube exit toward the ovule occurs. When NS silks were pollinated with NS pollen, pollen tubes were able to grow through the proximal region of the silk (region directly connected with the ovule; area a2 in Figure 3M) either with or without 4 h exposure to HS after pollination (Figures 3E and 3F). The same was observed when silks were exposed to 24 h HS before pollination and kept at control temperature after pollination (Figure S3C). In contrast, 37% (*n* = 30) silks exposed for 24 h to HS before pollination and an additional 4 h HS after pollination lacked pollen tubes at the proximal region at 24 h after pollination (HAP) (Figures S3C and S3D). To further analyze whether this is caused by delayed pollen tube growth or arrest, later time points at 27 HAP and 30 HAP were also investigated. Significant differences compared with pollen tubes at 24 HAP could not be observed (Figure 3N). Notably, 94.4% (*n* = 18) silks exposed for 48 h to HS before pollination and then kept at control temperature after pollination showed pollen tube growth at the proximal region at 24 HAP (Figure 3G), which is comparable with NS silks and silks exposed for 24 h to HS before pollination. However, only 68.4% (*n* = 19) silks exposed to 48 h HS before pollination and an additional 4 h after pollination showed lack of pollen tube growth at the proximal region (Figure 3H). At 27 HAP and 30 HAP, respectively, still 57% (*n* = 28) and 56% (*n* = 25) of silks lacked pollen tubes at the proximal region (Figure 3N). These findings indicate that HS on silks before and after pollination causes late growth arrest of pollen tubes.

To gain more insight into the cause of sterility, we also investigated micropylar pollen tube growth and guidance, which is the next critical step after late growth of pollen tube through the silk. At 24 HAP, aniline blue staining showed pollen tubes that penetrated through the micropylar ovule nucellus region into the female gametophyte (area a3 in Figure 3M) in 90.9% (*n* = 11) ovules at NS conditions before and after pollination (Figure 3J). Similarly, 90% (*n* = 10) ovules at NS condition before pollination and 4 h HS after pollination showed pollen tube growth at 24 HAP in the female gametophyte (Figure 3K). When silks were exposed to 24 h and 48 h HS before pollination, respectively, and then kept at NS conditions after pollination; 85.7% (*n* = 7) and 87.5% (*n* = 16) ovules (about 86%), respectively, showed pollen tube growth inside the female gametophyte (Figures 3K and S3E). However, only 28.6% (*n* = 7) ovules showed pollen tubes in this area when plants were exposed to 24 h HS before and 4 h HS after pollination. Moreover, none of the observed ovules (0%; *n* = 13) showed pollen tube growth into the female gametophyte after 48 h HS before and 4 h HS after pollination (Figures 3L and S3F). Altogether, these findings demonstrate that only the combination of heat stress before pollination and during pollen tube growth causes late pollen tube growth arrest, resulting in fertilization failure and sterility due to the lack of pollen tubes reaching the female gametophyte.

### ROS levels are increased in silks under heat stress and can be reduced by scavengers

**Figure 4:**
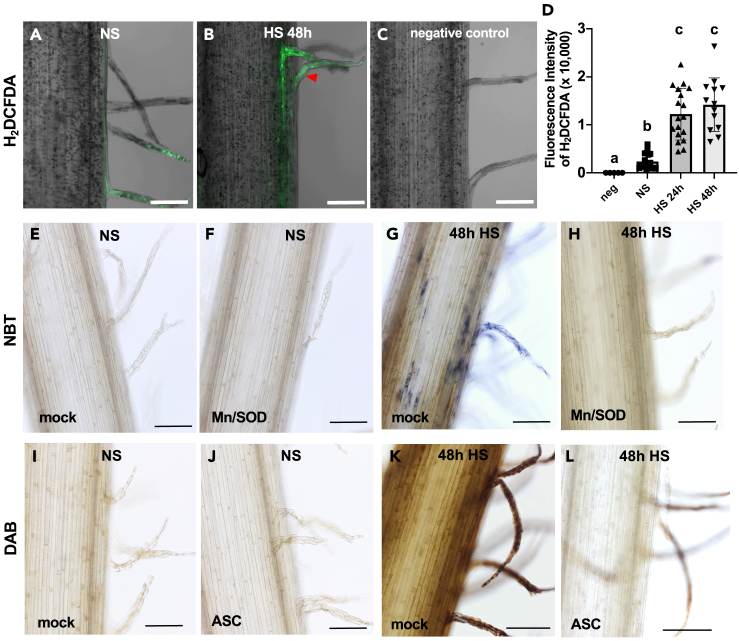
ROS levels increase in maize silks under heat stress, and ROS scavengers reduce their levels (A–C) Confocal microscopy of silks treated with H_2_DCFDA fluorescent probes at NS conditions (A) and after 48 h HS (HS 48 h) (B). Non-stressed silks with no probe was taken as negative control (C). Scale bars: 100 μm. (D) Quantification of relative fluorescence intensity of silks with a H_2_DCFDA probe. Data are presented as mean ± SD. Letters indicate significance categories. *p* < 0.01 by one-way ANOVA and the Tukey-Kramer test. (E–H) DIC microscopy of nitroblue tetrazolium (NBT) stained silks sprayed with mock solution (E, G) or sprayed with MnCl_2_ and SOD solution (F, H) during HS treatment. Silks (3 days after emergence) were kept at NS conditions (E, F) or under HS for 48 h (HS 48 h) (G, H). (I–L) DIC microscopy of 3,3′-diaminobenzidine (DAB)-stained silks sprayed with mock solution (I, K) or ascorbic acid (ASC) solution (J, L) during HS treatment. Silks (3 days after emergence) were kept at NS conditions (I, J) or under HS for 48 h (K, L). Scale bars: 100 μm.

ROS were reported to trigger cell death programs at high concentrations, whereas moderate ROS levels play critical roles in signal perception and transduction especially during environmental stress responses and reproduction in plants.^37^^,^^53^^,^^54^ Less is known about the role of ROS during reproduction in maize. A recent report showed that high ROS levels damage sperm cell DNA, which can be used for haploid induction,^55^ whereas overaccumulation of ROS during early kernel development leads to severe damage of nucellus and endosperm cells.^56^ To visualize the effect of HS on ROS levels in silks, first a general ROS detection probe (H_2_DCFDA) was used, which is fluorescent when oxidation occurs within a cell. Weak fluorescence signals could be detected in silk hairs and barely inside the silk at NS condition (Figure 4A). However, the fluorescent signal significantly increased in both silk hairs and silks after 24 h and 48 h HS exposure. Strongest signals were observed in the epidermal cell layer of the silk and silk hairs (Figures 4B and 4D). A significant difference in fluorescent signal intensity between 24 h and 48 h HS exposure could not be observed (Figure 4D). This indicates that ROS levels in silks significantly increase during HS.

To discriminate between ROS, NBT (nitroblue tetrazolium) staining was used to indicate O₂^·−^ (superoxide) levels. An increase of blue NBT stain was detected in silks and their silk hairs after 24 h and 48 h HS exposure, respectively (Figures 4E, 4G, and S4A). DAB (3,3′-diaminobenzidine) staining was used to indicate H₂O₂ levels. Again, an increase of DAB stain was observed in silks and silk hairs after HS (Figures 4I, 4K, and S4D). Taken together, both superoxide and H₂O₂ level were strongly increased in silks exposed to HS.

To visualize and quantify the role of increased ROS levels caused by HS during fertilization, we sprayed silks with ROS scavengers to reduce ROS levels. MnCl_2_ (Mn) together with superoxide dismutase (SOD) is a scavenger for O₂^·−^. As shown in Figures 4E–4H and S4A–S4C, NBT staining significantly decreased in silks and their hairs sprayed with Mn/SOD solution under HS, compared with the mock (water) control. On the other hand, DAB staining also decreased in silks sprayed with Mn/SOD solution under HS, compared with the mock control (Figures S4L–S4N). Ascorbic acid (ASC) is a scavenger for H₂O₂. In contrast to the mock control, after ASC spraying DAB staining significantly decreased in silks and silk hairs exposed to continuous HS (Figures 4I–4L and S4D–S4F). Both, Mn/SOD and ASC treatments decreased the fluorescent intensity of the ROS probe H_2_DCFDA (Figures S4G–S4K). These experiments demonstrate that superoxide scavenger SOD and H₂O₂ scavenger ASC significantly reduce ROS levels in silks caused by HS.

### ROS scavengers restore pollen tube penetration in silks under heat stress

**Figure 5:**
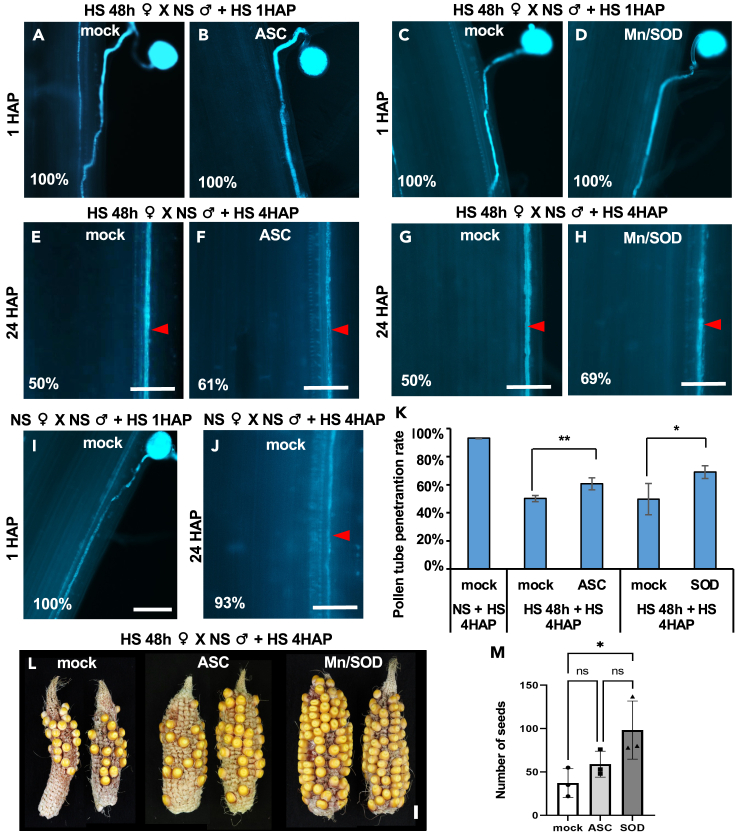
ROS scavengers restore pollen tube growth defects and sterility caused by heat stress (A–J) Fluorescent microscopy of aniline-blue-stained pollinated silks with indicated stress conditions and ROS scavenger treatment. For observed silk areas see Figure 3M. Percentages of observed phenotypes are indicated. (A–D) Silks (3 days after emergence) were treated with 48 h HS (HS 48 h ♀) and pollinated with NS pollen (♂), then treated with 1 h HS after pollination (+ HS 1HAP). Pollen tube early growth in the transmitting tract was observed 1 h after pollination (1 HAP). (E–H) Pollinated silks were treated with 4 h HS (+ HS 4HAP). The 1 to 2 cm proximal region (abscission zone) of silks was observed 24 h after pollination (24 HAP) for pollen tube late growth. Mock solution and ascorbic acid (ASC) solution were sprayed to silks during HS treatments (A, B, E, F). Mock solution and MnCl_2_ with SOD solution were sprayed to silks during HS (C, D, G, H). (I–J) NS silks pollinated with NS pollen and sprayed with mock solution served as a positive control for pollen tube growth at 1 HAP (I) and 24 HAP (J). Scale bars: 100 μm. (K) Percentage of silks showing pollen tube growth at the distal region (a2 in Figure 3M) was quantified at 24 HAP. Data are presented as mean ± SD. (L) Cobs harvested from indicated treatments. Representative examples of cobs after spraying mock, ASC, and MnCl_2_/SOD solution during the heat stress treatment. Scale bar: 1 cm. (M) Quantification of kernel (seeds) numbers from indicated treatments. ns: no significant difference. Data are presented as mean ± SD. One asterisk indicates *p* < 0.05 as analyzed by one-way ANOVA and the Tukey-Kramer test.

We next addressed the question whether HS-induced pollen tube late growth arrest is caused by increased ROS levels. We sprayed silks exposed for 48 h to HS with the H_2_O_2_ and O₂^·−^ scavengers ASC and Mn/SOD, respectively, and their corresponding mock controls. None of the spray treatments affected pollen germination and pollen tube growth into the transmitting tract (Figures 5A–5D). However, both ASC and Mn/SOD partially restored late pollen tube growth arrest as the number of pollen tubes observed in the proximal region of the silk (region a2 in Figure 3M) increased from 50% to 61% and to 69%, respectively, (Figures 5E–5H and 5K). NS silks (sprayed with the mock control for 48 h) pollinated by NS pollen and exposed to HS for 4 h showed normal (93%) pollen tube germination and growth (Figures 5I and 5J). Taken together, superoxide and H₂O₂ scavengers reduce ROS level caused by HS in maize silks and enable pollen tubes to grow longer distances. We further analyzed seed set of cobs harvested from silks exposed for 48 h HS after mock, ASC, and Mn/SOD spraying, respectively. Seed set of cobs sprayed with Mn/SOD significantly restored seed set and thus yield compared with mock-treated ones (Figures 5L and 5M). This indicates that reduction of HS-induced ROS levels in maize silks significantly improves fertility.

### Transcriptome analyses show downregulation of catabolic ROS genes and identification of transcription factors regulated in heat-stress-exposed silks

**Figure 6:**
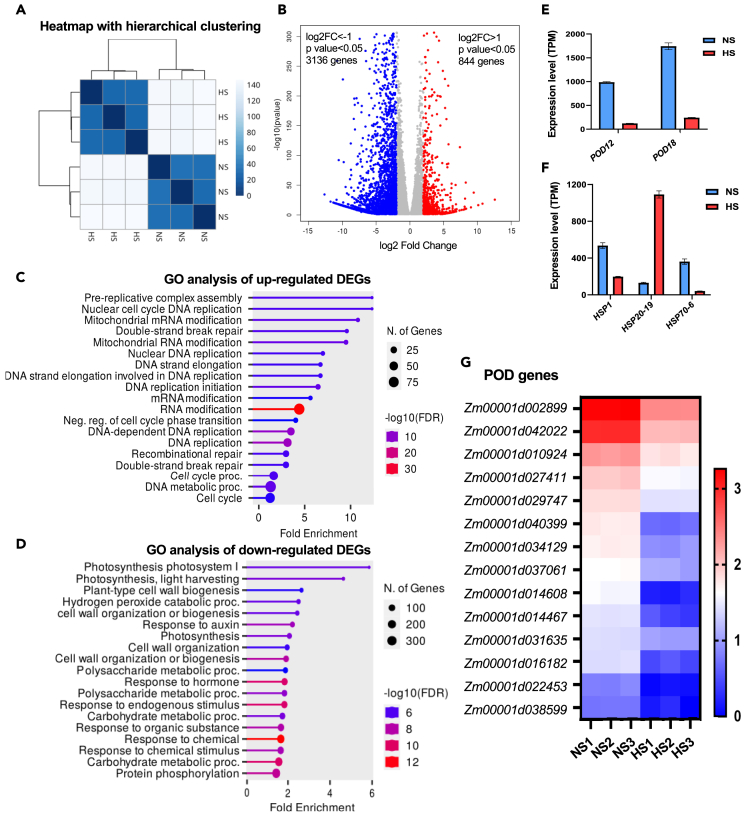
Expression analysis of genes in maize silks in response to heat stress (A) Heatmap with hierarchical clustering showing the distance between samples. (B) Volcano plot indicating differentially expressed genes (DEGs). Red and blue dots mark down- and upregulated genes, respectively, with *p* value <0.05, log2FC > 1, and log2FC < −1. (C) Gene Ontology (GO) biological process enrichment of significantly upregulated genes. (D) GO biological process enrichment of significantly downregulated genes. (E) Gene expression levels (TPM) of two most differentially expressed peroxidase (POD) genes. *POD12*: *Zm00001d042022*; *POD18*: *Zm00001d002899*. Data are presented as mean ± SD. (F) Gene expression levels (TPM) of three most differentially expressed heat shock protein (HSP) genes. (G) Heatmap of the expression level of peroxidase (POD) genes in HS and NS samples. The numbers on the color scale indicate the Log10 values of TPM.

To elucidate the molecular mechanisms of HS on maize silks, we performed RNA-seq analyses using the maize inbred line B73. Plants were exposed to moderate HS (35°C/25°C) for 48 h. Only silk regions directly exposed to the environment were harvested for sequencing. Each three biological replicates were sampled for NS and HS conditions. On average, 23 million reads were generated from each of the six samples (Table S1). Reads were mapped to the B73 maize reference genome (Ref_Gen4) downloaded from MaizeGDB (www.maizrgdb.org) with an average mapping rate of 90%. Normalized transcription level was calculated as transcript per million (TPM). On average, 19,500 expressed genes were detected in each sample (TPM>1) (Table S1). Principal-component analysis (PCA) and heatmap of sample-to-sample distance showed that gene expressions pattern after HS and NS control conditions were well separated. The three replicates of each condition were well clustered (Figures 6A and S5) and the transcriptome of all samples used for further studies.

We next identified differentially expressed genes (DEGs) (genes with TPM>1) of the HS condition based on the criteria |log2(foldchange)| >1 and *p* value < 0.05. Eight hundred forty-four upregulated and 3,136 downregulated genes were detected (Figure 6B). Gene ontology (GO) enrichment analysis of upregulated DEGs showed that most highly enriched biological processes are related to the cell cycle, DNA replication, and RNA modification, indicating that these pathways are positively influenced by moderate HS (Figure 6C). GO enrichment analysis for downregulated DEGs shows that most highly enriched biological processes are associated to photosynthesis, cell wall, and carbohydrate metabolism, indicating changes in basic metabolic pathways (Figure 6D). In addition, responses to hormones are also enriched, which could be expected from previous findings.^57^^,^^58^^,^^59^ Importantly, hydrogen peroxide catabolic processes are among the highly enriched downregulated DEGs, which is consistent with the increased ROS levels detected during heat stress responses (Figure 6D). Two highly expressed peroxidase (POD) genes *Zm00001d002899* (*POD12*) and *Zm00001d042022* (*POD18*) at NS conditions are strongly downregulated during HS (Figures 6E and 6G). In total, we detected 35 expressed *POD* genes in our samples, of which 27 *POD* genes are significantly downregulated in HS silks (Figure 6G). Reduction of peroxidase activity could explain the increase of ROS levels during HS described earlier.

Since Ca^2+^ signaling and ROS play critical roles in stress stimuli perception and transduction,^53^^,^^60^ we also investigated genes involved in Ca^2+^ signaling and ROS scavenging. Three genes encoding Ca^2+^-dependent protein kinases (CDPKs), *CDPK19* (*Zm00001d021835*), *CDPK22* (*Zm00001d015100*), and *CDPK36* (*Zm00001d027480*), and one gene encoding glutathione S-transferase (GST), *GST48* (*Zm00001d043795*), are downregulated (Figures S6C, S7A, and S7B), whereas the expression levels of genes encoding SODs are not significantly changed (Figure S6B). Heat shock proteins (HSPs) are major functional proteins for cellular homeostasis, protein conformation folding, and stabilization under stress conditions. A total of 12 *HSP* genes are differentially expressed (Figure S8A). Among the highly expressed *HSP* genes in silk tissue, only *HSP20-19* is strongly upregulated, whereas *HSP1* and *HSP70-6* are significantly downregulated (Figure 6F). Enzymes involved in ROS degradation are catalases, heme-containing enzymes that convert hydrogen peroxide to water and oxygen.^61^ Among three maize catalase genes, *ZmCAT1* (*Zm00001d014848*) and *ZmCAT2* (*Zm00001d027511*) are upregulated (Figure S9A). A previous study showed that botryoid pollen 1 (bp1) regulates ROS-triggered PCD by controlling uridine diphosphate (UDP)-sugar homeostasis in rice.^62^ We found that *hin3*, the homologous gene of *bp1*, is also downregulated (Figure S9B). Other genes previously shown to be associated with stress responses encode metallothionein (MT) proteins, which are small, cysteine-rich proteins that play important roles in plant growth and development, and are regulated during stress responses.^63^^,^^64^^,^^65^ Among nine maize *MT* genes, *ZmMT8* (*Zm00001d039914*) and *ZmMT9* (*Zm00001d048611*) are highly upregulated. Notably, *ZmMT9* is already strongly expressed in un-stressed silks (TPM>5000) (Figure S9C).

**Figure 7:**
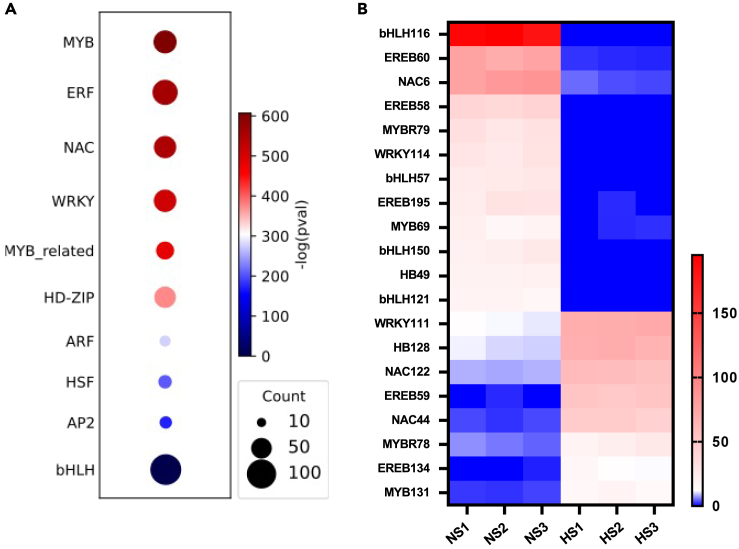
Enrichment of transcription factor genes in maize silks exposed to heat stress (A) Bubble chart showing significantly enriched TF families in response to heat stress. The most strongly induced family is shown at the top and the most strongly downregulated one at the bottom of the chart, respectively. (B) Heatmap of the expression level of the top 20 differentially expressed TF genes (Log2FC > 2 or Log2FC < −2, average TPM>10). The numbers on color scale indicate the TPM values.

Finally, we analyzed the expression level of transcription factor (TF) genes in silk tissue after HS expose. TFs including heat shock factors (HSFs) WRKY, MYB, NAC, bZIP, AP2/ERF, DREB, and bHLH play important roles in regulating gene expression of multiple genes in response to stress conditions.^66^^,^^67^^,^^68^^,^^69^ Seven HSF genes are differentially expressed (Figure S8B). Enrichment studies of TF families showed that in addition to genes encoding HSFs, genes for MYB, ERF, NAC, WRKY, MYB-related, HD-ZIP, ARF, AP2, and bHLH transcription factor families are enriched after HS (Figure 7A). Notably, many *bHLH* genes are among the top downregulated DEGs (10 of the 37 top downregulated TF genes; Table S3). Among them, *Zm00001d024522* (*bHLH116*), *Zm00001d043706* (*bHLH57*), *Zm00001d020705* (*bHLH150*), and *Zm00001d006065* (*bHLH121*) are highly downregulated (Log2FC < −9), whereas a number of NAC genes (4 of the 13 top upregulated TF genes) including *Zm00001d028999* (*NAC44*) and *Zm00001d022424* (*NAC122*) are among the top upregulated (Log2FC > 6) DEGs (Figure 7B and Table S3). This finding indicates that members of the same TF family are coordinately involved in HS responses but families partly act in opposite directions.

## Discussion

Due to global warming and associated risks to maintain or even increase agricultural seed production, research efforts to understand the effects of HS during the reproductive phase in maize and other crops has significantly increased in recent years. It is obvious that successful reproduction is the key to achieve full seed set and thus yield, but it has also been realized that the occurrence of high temperature often coincides with the reproduction phase as shown for maize and that could strongly limit yield.^18^^,^^70^ It has been shown that especially pollination and pollen development are highly susceptible to HS.^13^^,^^19^^,^^20^^,^^23^^,^^24^^,^^25^^,^^71^^,^^72^ In contrast to the amount of work carried out on pollen, little is known about the effect of HS on female reproductive organs, especially on silks that are directly exposed to ambient temperatures. Also inspired by discussions with maize farmers who suspected that silks play a key role for HS-induced sterility, we could confirm that plants exposed to HS during the silking and pollination stage show severe sterility, which is also consistent with previous findings.^30^^,^^31^ However, whether the silk tissue itself, the pollination process, or pollen tube growth as well as pollen tube reception are affected remained unknown together with the underlying molecular mechanisms.

Our detailed studies showed that HS-induced death of up to 50% hair cells at the silk surface did not affect pollen tube penetration and seed set probably due to multiple possibilities of pollen tubes to penetrate silk hair structures and the fact that the number of silk hairs and pollen are excessive for fertilization of each ovule. Moreover, silks exposed for 24 or even 48 h to HS before pollination did not lower seed setting significantly, but extending HS for only an additional 1–4 h after pollination led to dramatic seed set reduction. HS for 1–4 h HS after pollination without prior stress showed almost full seed set, indicating that both stressed silks for longer periods and additional stress during the pollen tube growth phase are responsible for observed sterility. The finding that HS leads especially to late pollen tube growth arrest suggests that, for example, communication between the pollen tube and the female transmitting tract tissue is affected, and HS may lead to structural changes of transmitting tract cells and/or the pollen tube cell wall (causing, e.g., cell wall stability/integrity defects) and insufficient energy and metabolite supply for high-speed pollen tube growth etc., ultimately leading to growth arrest. The observation that genes required for these processes belong to the strongest downregulated categories points toward this direction. The accumulation of ROS can cause oxidative burst and stress, involving lipid peroxidation, protein damage, nucleotide degradation, and ultimately cell death.^73^ NBT is a colorimetric dye for O2·—, which enables the study of early steps in oxidative stress induction by different treatments and environmental conditions in plants.^74^ DAB is a colorimetric dye for H2O2, which enables the visualization of H2O2 level at tissue and cellular levels.^75^ Fluorescent probes (e.g., H_2_DCFDA) are more sensitive than colorimetric ones. They possess a better signal-to-noise ratio compared to optical detection, which makes them more straightforward for quantification.^73^ However, there are high abundance of endogenous fluorescent compounds in plant cells and the cross-sensitivity to cellular antioxidants that compete the probes for ROS.^76^^,^^77^ We found that the ROS levels increased in silks after HS by using both colorimetric and fluorescent ROS detection methods. It is also possible that, for example, ROS accumulation triggers cell death similar to observation in pollen-pistil and pollen-tube transmitting tract interaction that were reported to lead to self-incompatibility responses.^78^^,^^79^ As multifunctional signaling molecules, ROS mediate highly diverse developmental and stress responses.^80^ ROS accumulation, distribution, and their well-balanced gradients are important to control, for example, stemness and differentiation during development^81^ or whether lateral roots, root hair, and defense responses are initiated, respectively.^82^ The homeostasis of apoplastic ROS largely depends on their generation by plasma-membrane-localized NADPH oxidases (RBOHs) and their inactivation by cell-wall-localized superoxide dismutases (SODs) and peroxidases (PODs).^81^^,^^83^^,^^84^ Our study showed that ROS levels are significantly increased in silks during HS, and the application of ROS scavengers can strongly reduce their levels and partly rescue pollen tube growth arrest and reduction of seed set. Transcriptomic studies can explain this finding as most POD genes expressed in silks are strongly downregulated during HS, which would lead to ROS accumulation due to downregulation of ROS scavengers. bHLH proteins are the second largest subfamily of transcription factors in plants and involved in various biological processes, including abiotic stress responses.^85^ Previous studies showed that the overexpression of *PtrbHLH*, *AtbHLH112*, or *MdbHLH130* increases SOD or POD activity and reduces ROS accumulation under stress conditions.^86^^,^^87^ It will now be important to elucidate whether downregulation of PODs is caused by simultaneous downregulation of TF genes reported earlier like *bHLH57*, *bHLH108*, *bHLH116*, and *bHLH79,* respectively, and whether theses bHLHs can directly regulate expression of POD genes. The identification of downregulated *POD* and *bHLH* genes now opens exciting future approaches with the long-term goal to engineer HS tolerant maize.

Other genes downregulated after HS encode among others calcium-dependent protein kinases (CDPKs). Ca^2+^ signaling and responses are tightly associated with environmental stresses including HS.^60^ HS causes overaccumulating of ROS, which stimulates Ca^2+^ signaling pathways^88^ including phosphorylation and thus activation of CDPKs.^89^^,^^90^ Downregulation of genes encoding CDPKs probably inactivates certain downstream signaling pathways in silks that may be important to support pollen tube growth. *hin3*, the homolog gene of rice *botryoid pollen 1* (*bp1*), is downregulated after HS, which is in consistent with its postulated function in preventing PCD caused by overaccumulation of ROS.^62^ This suggests that UDP-sugar homeostasis may also play a role in HS response in maize silks. Metallothioneins are small, ubiquitous Cys-rich proteins known to be involved in ROS scavenging and metal homeostasis.^65^ We found that *ZmMT8* and *ZmMT9* are highly upregulated, indicating a positive response to HS. Some of the ROS scavenger genes (like *POD* genes) are downregulated after HS, whereas other genes (like *MT* genes) are upregulated, suggesting different ROS scavenging pathways may act independently in response to HS.

Moreover, silk tissue was indeed reported to be more susceptible to HS than vegetative tissues in regard of restoring ROS levels.^53^ As a consequence, the tissue undergoes cell death instead of becoming heat tolerant. More detailed studies are now required to understand the interaction of ROS and Ca^2+^ signaling during pollen tube growth inside the silk and especially in the proximal silk region where growth of pollen tubes is arrested during HS. Notably, some NAC TF genes are highly upregulated in this silks region during aging that include *KIRA1-LIKE1* (*KIL1*; *NAC36*) and *KIL2-5 (NAC65*, *17*, *51* and *104*), of which KIL1 was recently shown to promote senescence and PCD in the proximal region of the silk.^51^ Loss of KIL1 function was shown to increase kernel yield following late pollination. It will now be important to elucidate whether loss of HS-induced NAC TFs can also result in decreased cell death and sensitivity of HS during the silking stage and may lead to yield increases. In summary, this study elucidated the most critical stage during the pollination process in maize, resulting in sterility during HS application. It further identified signaling pathway components and TFs that could potentially be used in the future to engineer crops avoiding sterility caused by HS during the pollination phase.

### Limitations of the study

Our experiments were done in the greenhouse and highly controllable walk-in growth chambers. The numbers of maize cobs collected to quantify the effect of heat stress was therefore limited, and we could not simulate field conditions. Heat stress in this study was only applied selectively to the female side; in the field, the effect of heat stress occurs on both male and female flower organs at the same time. ROS scavengers to restore pollen tube growth were directly applied to silks; consequences of spraying scavengers to whole plants were not investigated. Transgenic approaches to overexpress and knock out identified candidate genes to engineer tolerant maize plants is a long-term goal and beyond the scope of this study.

## STAR★Methods

### Key resources table

**Table:** 

REAGENT or RESOURCE	SOURCE	IDENTIFIER
NBT staining solution	0.1 mg/mL nitro-blue tetrazolium chloride in 25 mM HEPES buffer, pH 7.6	Thermo Fisher, 34035
DAB staining solution	0.1 mg/mL 3,3’-diaminobenzidine tetrahydrochloride hydrate in 50 mM tris-acetate buffer, pH 5.0	Sigma-Aldrich, D5637
ASC solution	10 mM L-ascorbic acid in water	Sigma-Aldrich, A4403
SOD and MnCl_2_ solution	10 U/mL SOD in 10 mM MnCl_2_	Sigma-Aldrich, S9697

### Resource availability

#### Lead contact

Further information and requests for resources and reagents should be directed to the lead contact, Thomas Dresselhaus (thomas.dresselhaus@ur.de).

#### Materials availability

This study did not generate new plant materials. Requests for materials listed in the key resources table should be directed to the lead contact.

#### Data and code availability

• Raw reads were submitted to ENA (project accession PRJEB69216). The reference genome of maize (version 4) and the annotation file are available at Maize GDB (https://www.maizegdb.org/).
• All data including microscopic data reported in this paper will be shared by the lead contact on request.
• Any additional information required to reanalyze the data reported in this study is available from the lead contact on request.

### Experimental model and study participant details

#### Plant growth and heat stress conditions

Seeds of maize inbred line B73 were soaked in water overnight and then transferred into small pots (10 cm diameter, 1 seed per pot) and gown for about three weeks. Then seedlings were transferred into 10 L pots. Plants were grown in a greenhouse under controlled conditions of 16 hours of light at 26 ± 2°C and 8 hours of darkness at 21 ± 2°C. Air humidity was maintained at around 60-65% and additional 24,000 lux light was provided. Plants were watered by an automated temperature-water-based irrigation system, which based on plant consumption in a time-based pre-programmed schedule. Fertilizer was applied with 2% Hakaphos (Compo Expert) twice a week and monitored throughout the entire vegetative and reproductive development. Maize ears were covered with paper bags before silk emerging to avoid pollen contamination. For crossings, old pollen was removed from tassels in the morning. Two to three hours later, fresh pollens were used to pollinate covered ears. Afterwards bags were marked and kept on ears. Thirty days after pollination, plants were transferred into a dry chamber with lower humidity and ceased watering. Around three weeks later, cobs were harvested and dried.

Heat stress (HS) was applied 3 days after silk emergence and tassels were removed from plants used as pollen recipients. Plants were transferred to walk-in growth chambers. For heat stress, growth chamber day/night temperature conditions were set at 35° for 16 h light (25,000 lux) and 25°C darkness with 60% air humidity for 24 h and 48 h, respectively. Correspondingly, non-stressed (NS) plants were maintained at a 26°/21°C day/night temperature regime with 60% humidity at 25,000 lux in a control chamber and examined at the same silk stages. Control and HS treated plants were watered daily to exclude drought stress effects. After HS exposure, all plants were maintained at control conditions for further examination and harvesting RNA-seq samples.

### Method details

#### *In vivo* pollen tube growth observation

*In vivo* pollen germination was carried out in a greenhouse. Fresh pollens were collected with a paper bag and an excess of them applied on silks. To avoid pollen contamination, covered bags on cobs were only shortly removed during pollination. At different time points after pollination, pollinated silks were cut off and fixed in 9:1 v/v ethanol:acetic acid at 4°C overnight. *In vivo* pollen tube visualization via aniline blue staining was achieved according to the method of Marten (1959) with adjustments: fixed samples were rehydrated by a water series. Then they were treated by 8 M sodium hydroxide for 2 hours to clear and soften tissues. Softened silks were washed in 1x PBS (phosphate Buffered Saline) for three times. Staining was carried out in aniline blue solution (0.1% aniline blue; 0.1 M K_2_HPO_4_·3H_2_O, pH=11) overnight at 4°C. For ovule sections, a thin slice (1 mm) containing the embryo sac with the micropylar region was cut by two sharp blades and proceeded with the same steps for aniline blue staining. Specimens were washed and mounted with fresh staining solution on a slide with a cover slip and imaged using a fluorescence microscope ZEISS Axio Imager 2 with a 20x objective (Plan-Apochromat 20x/0.8 M27) at 350∼400nm (UVA) excitation.

#### ROS staining

For superoxide detection, silks were cut and stained in 0.1 mg/mL NBT (Nitroblue Tetrazolium) solution (see key resources table) and incubated at room temperature in the dark for 2 h. For superoxide scavenger treatment, 10 U/mL SOD with 10 mM MnCl_2_ solution was sprayed on silks three times a day. As a mock control, the same amount of water was sprayed on silks three times a day. For H_2_O_2_ staining, silks were cut and stained in 0.1 mg/mL DAB (3,3′-Diaminobenzidine) solution (see key resources table) and incubated at room temperature in the dark for 8 h. For H_2_O_2_ scavenger treatment, 10 mM ascorbate (ASC) solution (see key resources table) was sprayed on silks three times a day. As mock control, the same amount of water was sprayed on silks also three times a day. To remove pigments of silks for better imaging, stained silks were transferred to 80% ethanol and incubated at 70°C for 10 min.

For ROS staining, the fluorescent dye CM-H_2_DCFDA (Invitrogen, C6827) was used. CM-H_2_DCFDA was dissolved in DMSO (dimethyl sulfoxide) as stock solution. Silks were cut and incubated using a working concentration of 1 μM CM-H_2_DCFDA in 1x PBS solution in the dark for 10 minutes and then washed two times with PBS. A Zeiss LSM980 Airyscan confocal microscope using a 10x objective and excitation at 488 nm was used to take images.

#### TUNEL assay

The TUNEL assay was perfomed as follows: first, silks were fixed for 1h in 4% (v/v) paraformaldehyde in PBS under vacuum at room temperature. Then samples were washed three times in PBS and kept for 2 min on ice with 0.1% Triton X-100 in 0.1% sodium citrate followed by three times PBS washing. As a positive control, fixed samples were treated with DNase I for 15 min and thereafter washed three times with PBS. The TUNEL reaction was performed in 1.5 mL tubes and each time 100 μL label solution removed as a negative control. 50 μL enzyme solution was added to the remaining 450 μL label solution to obtain a 500 μL TUNEL reaction mixture that was incubated with samples at 37°C for 60 min in the dark. Then samples were washed three times with PBS and counterstained with 1 μg/mL DAPI. For TUNEL imaging, a Zeiss LSM980 Airyscan confocal microscope was used with a 20x objective and excitation/emission at 488/655 nm.

#### RNA-seq and bioinformatic analyses

Library preparation and RNAseq were carried out as described in the Illumina TruSeq Stranded mRNA Sample Preparation Guide, the Illumina NextSeq 2000 Sequencing System Guide (Illumina Inc.), and the KAPA Library Quantification Kit-Illumina/ABI Prism (Roche Sequencing Solutions Inc.). Sequencing was performed using the Illumina NextSeq 2000 Sequencing System. Libraries were generated from 6 samples (three replicates of a silk control and three replicates of silks exposed for 48 h to heat stress) and sequenced. On average around 23 million paired-end 130 bp reads were obtained for each library. Raw reads were submitted to ENA. Project accession is PRJEB69216.

The quality of raw sequencing reads in FastQ format was examined by FastQC.^91^ Low-quality reads were removed and remaining ones trimmed to remove adapter sequences using Trimmomatic v0.39 (Bolger et al., 2014). Reads were quality filtered and trimmed using the following settings: LEADING:3, SLIDINGWINDOW:4:15, MINLEN:40. HISAT2^92^ was used to align high-quality reads to the reference maize genome (B73 release RefGen_v4). After alignment, featureCounts was used to identify number of reads that mapped to genes.^93^ Differential gene expression (DEG) analysis was performed using the R package DESeq2.^94^ DESeq2 uses the output of featureCounts to quantify changes in expression levels by estimating fold change (FC) in gene expression between different treatment groups. Default parameters were used as provided from the DESeq2 package. Genes showing a |log2FC| >1 and adjusted p-value<0.05 were considered differentially expressed genes (DEGs) and were identified through fold change filtering. Heatmaps were generated using TPM values of selected genes in Grapfpad Prism. Valcano plots were generated by using the online tool Galaxy.^95^ The expression heatmap visualizes sample-to-sample distances derived from the variance-stabilizing transformation of count data, representing overall gene expression patterns. The distances were computed using the "pheatmap" package in R, incorporating hierarchical clustering for both rows and columns. The resulting heatmap provides a concise representation of similarities and differences in gene expression profiles among samples, offering insights into the broader patterns of gene expression variation within the dataset. Gene Ontology (GO) enrichment studies were performed via the enrichment analysis function of R package BioNERO using Gene Ontology (GO) annotations available within MaizeMine.^96^ This function uses Fisher’s exact test (Graham J. G. Upton 1992) as a statistical method to test for over-representation and return a set of enriched GO terms. In a similar way transcription factor (TF) families enrichment was perfomed by comparing the occurrence of TF families in different gene sets to identify TF families that were significantly enriched. To establish the background, the occurrence frequency of each TF family across all genes was determined. For sample frequency, the occurrence within a list of differentially expressed genes (DEGs) associated with each sample was considered.

### Quantification and statistical analysis

Microscopy images: Individual treatment of images on a given figure was never performed to enable comparison between patterns/intensities.

Figures 1 and S1: Confocol images in Figures 1A, 1B, and S1A–S1C are representatives of numerous observations on more than the individual plants each (numbers of samples observed are written on each image). Significantly associated groups are marked by letters, calculated by Tukey-Kramer test with *p* < 0.01 (Figures 1E, 1F, and S1D).

Figure 2: Numbers of cobs quantified are written below each box. Significantly associated groups are indicated by letters, calculated by two-way ANOVA and the Tukey test.

Figures 3 and S3: Confocol images in Figures 3A–3L and S3A–S3F are representative for numerous observations on more than 3 individual plants each (numbers of samples observed are written on each image). The quantification in Figure 3N is based on 3 individual plants.

Figure 4: Confocol images in Figures 4A–4C are representatives of numerous observations on more than 3 individual plants each. The fluoresence intensity was quantified using Zeiss Zen Blue 3.4 and the significance analyzed by the one-way ANOVA-TUKEY test.

Figure 5: Confocol images in Figures 5A–5J are representatives of numerous observations on more than 3 individual plants each. The quantifications in Figures 5K and 5M are from 3 cobs each, and significance analyzed by the one-way ANOVA-TUKEY test.

Figure 6: The bar graphs in Figures 6E and 6F and the heatmap in Figure 6G are generated with absolut and Log10 values of TPMs using GraphPad Prism 9.3.1.

## References

[1] Wahid A., Gelani S., Ashraf M., Foolad M. (2007). Heat tolerance in plants: An overview. Environ. Exp. Bot..

[2] Cairns J.E., Sonder K., Zaidi P.H., Verhulst N., Mahuku G., Babu R., Nair S.K., Das B., Govaerts B., Vinayan M.T. (2012). Maize production in a changing climate: Impacts, adaptation, and mitigation Sstrategies. Adv. Agron..

[3] Lobell D.B., Asner G.P. (2003). Climate and management contributions to recent trends in US agricultural yields. Science.

[4] Lobell D.B., Bänziger M., Magorokosho C., Vivek B. (2011). Nonlinear heat effects on African maize as evidenced by historical yield trials. Nat. Clim. Chang..

[5] Lobell D.B., Hammer G.L., McLean G., Messina C., Roberts M.J., Schlenker W. (2013). The critical role of extreme heat for maize production in the United States. Nat. Clim. Chang..

[6] Hawkins E., Fricker T.E., Challinor A.J., Ferro C.A.T., Ho C.K., Osborne T.M. (2013). Increasing influence of heat stress on French maize yields from the 1960s to the 2030s. Glob. Chang. Biol..

[7] Kumari P., Wani I.A., Khan S., Verma S., Mushtaq S., Gulnaz A., Paray B.A. (2022). Modeling of Valeriana wallichii habitat suitability and niche dynamics in the Himalayan region under anticipated climate change. Biology.

[8] Meehl G.A., Tebaldi C. (2004). More intense, more frequent, and longer lasting heat waves in the 21st century. Science.

[9] Marx W., Haunschild R., Bornmann L. (2021). Heat waves: a hot topic in climate change research. Theor. Appl. Climatol..

[10] Klingelhofer D., Braun M., Bruggmann D., Groneberg D.A. (2023). Heatwaves: does global research reflect the growing threat in the light of climate change?. Global Health.

[11] Lobell D.B., Schlenker W., Costa-Roberts J. (2011). Climate trends and global crop production since 1980. Science.

[12] Zhu P., Zhuang Q., Archontoulis S.V., Bernacchi C., Müller C. (2019). Dissecting the nonlinear response of maize yield to high temperature stress with model-data integration. Glob. Chang. Biol..

[13] Chaturvedi P., Wiese A.J., Ghatak A., Záveská Drábková L., Weckwerth W., Honys D. (2021). Heat stress response mechanisms in pollen development. New Phytol..

[14] Giorno F., Wolters-Arts M., Mariani C., Rieu I. (2013). Ensuring reproduction at high temperatures: The heat stress response during anther and pollen development. Plants.

[15] Saini H.S., Sedgley M., Aspinall D. (1983). Effect of heat-stress during floral development on pollen-tube growth and ovary anatomy in wheat (Triticum aestivum L.). Funct. Plant Biol..

[16] Zenda T., Wang N., Dong A., Zhou Y., Duan H. (2022). Reproductive-stage heat stress in cereals: Impact, plant responses and strategies for tolerance improvement. Int. J. Mol. Sci..

[17] Tao Z.Q., Chen Y.Q., Zou J.X., Li C., Yuan S.F., Yan P., Shi J.T., Sui P. (2016). Spectral characteristics of spring maize varieties with different heat tolerance to high temperature. Spectrosc. Spect. Anal..

[18] Ma X., Su Z., Ma H. (2020). Molecular genetic analyses of abiotic stress responses during plant reproductive development. J. Exp. Bot..

[19] Begcy K., Nosenko T., Zhou L.Z., Fragner L., Weckwerth W., Dresselhaus T. (2019). Male sterility in maize after transient heat stress during the tetrad stage of pollen development. Plant Physiol..

[20] Wang Y., Tao H., Tian B., Sheng D., Xu C., Zhou H., Huang S., Wang P. (2019). Flowering dynamics, pollen, and pistil contribution to grain yield in response to high temperature during maize flowering. Environ. Exp. Bot..

[21] Liu X., Wang X., Wang X., Gao J., Luo N., Meng Q., Wang P. (2020). Dissecting the critical stage in the response of maize kernel set to individual and combined drought and heat stress around flowering. Environ. Exp. Bot..

[22] Bheemanahalli R., Ramamoorthy P., Poudel S., Samiappan S., Wijewardane N., Reddy K.R. (2022). Effects of drought and heat stresses during reproductive stage on pollen germination, yield, and leaf reflectance properties in maize (Zea mays L.). Plant Direct.

[23] Li X., Bruckmann A., Dresselhaus T., Begcy K. (2024). Heat stress at the bicellular stage inhibits sperm cell development and transport into pollen tubes. Plant Physiol..

[24] De Storme N., Geelen D. (2014). The impact of environmental stress on male reproductive development in plants: biological processes and molecular mechanisms. Plant Cell Environ..

[25] Bheemanahalli R., Sunoj V.S.J., Saripalli G., Prasad P.V.V., Balyan H.S., Gupta P.K., Grant N., Gill K.S., Jagadish S.V.K. (2019). Quantifying the impact of heat stress on pollen germination, seed set, and grain filling in spring wheat. Crop Sci..

[26] Tsou C.H., Cheng P.C., Tseng C.M., Yen H.J., Fu Y.L., You T.R., Walden D.B. (2015). Anther development of maize (Zea mays) and longstamen rice (Oryza longistaminata) revealed by cryo-SEM, with foci on locular dehydration and pollen arrangement. Plant Reprod..

[27] Zhou L.Z., Juranić M., Dresselhaus T. (2017). Germline development and fertilization mechanisms in maize. Mol. Plant.

[28] Dresselhaus T., Lausser A., Márton M.L. (2011). Using maize as a model to study pollen tube growth and guidance, cross-incompatibility and sperm delivery in grasses. Ann. Bot..

[29] El-Sappah A.H., Rather S.A., Wani S.H., Elrys A.S., Bilal M., Huang Q., Dar Z.A., Elashtokhy M.M.A., Soaud N., Koul M. (2022). Heat stress-mediated constraints in maize (Zea mays) production: Challenges and solutions. Front. Plant Sci..

[30] Bassetti P., Westgate M.E. (1993). Emergence, elongation, and senescence of maize silks. Crop Sci..

[31] Borras L., Vitantonio-Mazzini L.N. (2018). Maize reproductive development and kernel set under limited plant growth environments. J. Exp. Bot..

[32] Bedinger P. (1992). The remarkable biology of pollen. Plant Cell.

[33] Dresselhaus T., Franklin-Tong N. (2013). Male-female crosstalk during pollen germination, tube growth and guidance, and double fertilization. Mol. Plant.

[34] Zhou L.Z., Dresselhaus T. (2019). Friend or foe: Signaling mechanisms during double fertilization in flowering seed plants. Curr. Top. Dev. Biol..

[35] Waszczak C., Carmody M., Kangasjärvi J. (2018). Reactive oxygen species in plant signaling. Annu. Rev. Plant Biol..

[36] Zhang M.J., Zhang X.S., Gao X.Q. (2020). ROS in the male-female interactions during pollination: Function and regulation. Front. Plant Sci..

[37] Zhou L.Z., Dresselhaus T. (2023). Multiple roles of ROS in flowering plant reproduction. Adv. Bot. Res..

[38] Cardenas L., McKenna S.T., Kunkel J.G., Hepler P.K. (2006). NAD(P)H oscillates in pollen tubes and is correlated with tip growth. Plant Physiol..

[39] Liu P., Li R.L., Zhang L., Wang Q.L., Niehaus K., Baluska F., Samaj J., Lin J.X. (2009). Lipid microdomain polarization is required for NADPH oxidase-dependent ROS signaling in Picea meyeri pollen tube tip growth. Plant J..

[40] Martin M.V., Fiol D.F., Sundaresan V., Zabaleta E.J., Pagnussat G.C. (2013). oiwa, a female gametophytic mutant impaired in a mitochondrial manganese-superoxide dismutase, reveals crucial roles for reactive oxygen species during embryo sac development and fertilization in Arabidopsis. Plant Cell.

[41] Lassig R., Gutermuth T., Bey T.D., Konrad K.R., Romeis T. (2014). Pollen tube NAD(P)H oxidases act as a speed control to dampen growth rate oscillations during polarized cell growth. Plant J..

[42] Xie H.T., Wan Z.Y., Li S., Zhang Y. (2014). Spatiotemporal production of reactive oxygen species by NADPH oxidase is critical for tapetal programmed cell death and pollen development in Arabidopsis. Plant Cell.

[43] Huang H., Ullah F., Zhou D.X., Yi M., Zhao Y. (2019). Mechanisms of ROS regulation of plant development and stress responses. Front. Plant Sci..

[44] Jimenez-Quesada M.J., Traverso J.A., Potocký M., Žárský V., Alché J.d.D. (2019). Generation of superoxide by OeRbohH, a NADPH oxidase activity during olive (Olea europaea L.) pollen development and germination. Front. Plant Sci..

[45] Völz R., Harris W., Hirt H., Lee Y.H. (2022). ROS homeostasis mediated by MPK4 and SUMM2 determines synergid cell death. Nat. Commun..

[46] Liu C., Shen L., Xiao Y., Vyshedsky D., Peng C., Sun X., Liu Z., Cheng L., Zhang H., Han Z. (2021). Pollen PCP-B peptides unlock a stigma peptide-receptor kinase gating mechanism for pollination. Science.

[47] Muhlemann J.K., Younts T.L.B., Muday G.K. (2018). Flavonols control pollen tube growth and integrity by regulating ROS homeostasis during high-temperature stress. Proc. Natl. Acad. Sci. USA.

[48] Møller I.M., Jensen P.E., Hansson A. (2007). Oxidative modifications to cellular components in plants. Annu. Rev. Plant Biol..

[49] Mittler R. (2002). Oxidative stress, antioxidants and stress tolerance. Trends Plant Sci..

[50] Gao Z., Daneva A., Salanenka Y., Van Durme M., Huysmans M., Lin Z., De Winter F., Vanneste S., Karimi M., Van de Velde J. (2018). KIRA1 and ORESARA1 terminate flower receptivity by promoting cell death in the stigma of Arabidopsis. Nat. Plants.

[51] Šimášková M., Daneva A., Doll N., Schilling N., Cubr A.R.O.M., Zhou L., De Winter F., Aesaert S., De Rycke R., Pauwels L. (2022). KIL1 terminates fertility in maize by controlling silk senescence. Plant Cell.

[52] Lausser A., Kliwer I., Srilunchang K.O., Dresselhaus T. (2010). Sporophytic control of pollen tube growth and guidance in maize. J. Exp. Bot..

[53] Li Y., Wang X., Li Y., Zhang Y., Gou Z., Qi X., Zhang J. (2020). Transcriptomic analysis revealed the common and divergent responses of maize seedling leaves to cold and heat stresses. Genes.

[54] Liu H., Song S., Zhang H., Li Y., Niu L., Zhang J., Wang W. (2022). Signaling transduction of ABA, ROS, and Ca(2+) in plant stomatal closure in response to drought. Int. J. Mol. Sci..

[55] Jiang C., Sun J., Li R., Yan S., Chen W., Guo L., Qin G., Wang P., Luo C., Huang W. (2022). A reactive oxygen species burst causes haploid induction in maize. Mol. Plant.

[56] Zhang K., Wang F., Liu B., Xu C., He Q., Cheng W., Zhao X., Ding Z., Zhang W., Zhang K., Li K. (2021). ZmSKS13, a cupredoxin domain-containing protein, is required for maize kernel development via modulation of redox homeostasis. New Phytol..

[57] Li N., Euring D., Cha J.Y., Lin Z., Lu M., Huang L.J., Kim W.Y. (2020). Plant hormone-mediated regulation of heat tolerance in response to global climate change. Front. Plant Sci..

[58] Muslu A.S., Kadioglu A. (2021). Role of abscisic acid, osmolytes and heat shock factors in high temperature thermotolerance of *Heliotropium thermophilum*. Physiol. Mol. Biol. Plants.

[59] Jing H., Wilkinson E.G., Sageman-Furnas K., Strader L.C. (2023). Auxin and abiotic stress responses. J. Exp. Bot..

[60] Jammes F., Hu H.C., Villiers F., Bouten R., Kwak J.M. (2011). Calcium-permeable channels in plant cells. FEBS J..

[61] Guan L., Scandalios J.G. (1995). Developmentally related responses of maize catalase genes to salicylic acid. Proc. Natl. Acad. Sci. USA.

[62] Chen H., Zhang S., Li R., Peng G., Chen W., Rautengarten C., Liu M., Zhu L., Xiao Y., Song F. (2023). BOTRYOID POLLEN 1 regulates ROS-triggered PCD and pollen wall development by controlling UDP-sugar homeostasis in rice. Plant Cell.

[63] Gao C., Gao K., Yang H., Ju T., Zhu J., Tang Z., Zhao L., Chen Q. (2022). Genome-wide analysis of metallothionein gene family in maize to reveal its role in development and stress resistance to heavy metal. Biol. Res..

[64] Kumar G., Kushwaha H.R., Panjabi-Sabharwal V., Kumari S., Joshi R., Karan R., Mittal S., Pareek S.L.S., Pareek A. (2012). Clustered metallothionein genes are co-regulated in rice and ectopic expression of OsMT1e-P confers multiple abiotic stress tolerance in tobacco via ROS scavenging. BMC Plant Biol..

[65] Wong H.L., Sakamoto T., Kawasaki T., Umemura K., Shimamoto K. (2004). Down-regulation of metallothionein, a reactive oxygen scavenger, by the small GTPase OsRac1 in rice. Plant Physiol..

[66] Schramm F., Larkindale J., Kiehlmann E., Ganguli A., Englich G., Vierling E., von Koskull-Döring P. (2008). A cascade of transcription factor DREB2A and heat stress transcription factor HsfA3 regulates the heat stress response of Arabidopsis. Plant J..

[67] Guan Q., Yue X., Zeng H., Zhu J. (2014). The protein phosphatase RCF2 and its interacting partner NAC019 are critical for heat stress-responsive gene regulation and thermotolerance in Arabidopsis. Plant Cell.

[68] Seo J.S., Joo J., Kim M.J., Kim Y.K., Nahm B.H., Song S.I., Cheong J.J., Lee J.S., Kim J.K., Choi Y.D. (2011). OsbHLH148, a basic helix-loop-helix protein, interacts with OsJAZ proteins in a jasmonate signaling pathway leading to drought tolerance in rice. Plant J..

[69] Surabhi G.-K., Badajena B. (2020). Transcription Factors for Abiotic Stress Tolerance in Plants.

[70] Tao Z.Q., Chen Y.Q., Li C., Zou J.X., Yan P., Yuan S.F., Wu X., Sui P. (2016). The causes and impacts for heat stress in spring maize during grain filling in the North China Plain - A review. J. Integr. Agric..

[71] Li Y.T., Xu W.W., Ren B.Z., Zhao B., Zhang J., Liu P., Zhang Z.S. (2020). High temperature reduces photosynthesis in maize leaves by damaging chloroplast ultrastructure and photosystem II. J. Agron. Crop Sci..

[72] Wang Y., Sheng D., Zhang P., Dong X., Yan Y., Hou X., Wang P., Huang S. (2021). High temperature sensitivity of kernel formation in different short periods around silking in maize. Environ. Exp. Bot..

[73] Ortega-Villasante C., Burén S., Blázquez-Castro A., Barón-Sola Á., Hernández L.E. (2018). Fluorescent *in vivo* imaging of reactive oxygen species and redox potential in plants. Free Radic. Biol. Med..

[74] Doke N. (1983). Generation of superoxide anion by potato-tuber protoplasts during the hypersensitive response to hyphal wall components of Phytophthora infestans and specific inhibition of the reaction by suppressors of hypersensitivity. Physiol. Plant Pathol..

[75] ThordalChristensen H., Zhang Z., Wei Y., Collinge D.B. (1997). Subcellular localization of H2O2 in plants. H2O2 accumulation in papillae and hypersensitive response during the barley-powdery mildew interaction. Plant J..

[76] Suzuki N., Koussevitzky S., Mittler R., Miller G. (2012). ROS and redox signalling in the response of plants to abiotic stress. Plant Cell Environ..

[77] Talamond P., Verdeil J.L., Conéjéro G. (2015). Secondary metabolite localization by autofluorescence in living plant cells. Molecules.

[78] Haque T., Eaves D.J., Lin Z., Zampronio C.G., Cooper H.J., Bosch M., Smirnoff N., Franklin-Tong V.E. (2020). Self-incompatibility triggers irreversible oxidative modification of proteins in incompatible pollen. Plant Physiol..

[79] Zhou L.Z., Qu L.J., Dresselhaus T. (2021). Stigmatic ROS: regulator of compatible pollen tube perception?. Trends Plant Sci..

[80] Ali S., Tyagi A., Bae H. (2023). ROS interplay between plant growth and stress biology: Challenges and future perspectives. Plant Physiol. Biochem..

[81] Qin Q. (2023). ROS: Important factor in plant stem cell fate regulation. J. Plant Physiol..

[82] Mase K., Tsukagoshi H. (2021). Reactive oxygen species link gene regulatory networks during Arabidopsis root development. Front. Plant Sci..

[83] Rajput V.D., Harish, Singh R.K., Verma K.K., Sharma L., Quiroz-Figueroa F.R., Meena M., Gour V.S., Minkina T., Sushkova S., Mandzhieva S. (2021). Recent developments in enzymatic antioxidant defence mechanism in plants with special reference to abiotic stress. Biology.

[84] Hu C.H., Wang P.Q., Zhang P.P., Nie X.M., Li B.B., Tai L., Liu W.T., Li W.Q., Chen K.M. (2020). NADPH Oxidases: The vital performers and center hubs during plant growth and signaling. Cells.

[85] Song L., Huang S.S.C., Wise A., Castanon R., Nery J.R., Chen H., Watanabe M., Thomas J., Bar-Joseph Z., Ecker J.R. (2016). A transcription factor hierarchy defines an environmental stress response network. Science.

[86] Huang X.S., Wang W., Zhang Q., Liu J.H. (2013). A basic helix-loop-helix transcription factor, PtrbHLH, of Poncirus trifoliata confers cold tolerance and modulates peroxidase-mediated scavenging of hydrogen peroxide. Plant Physiol..

[87] Zhao Q., Fan Z., Qiu L., Che Q., Wang T., Li Y., Wang Y. (2020). MdbHLH130, an apple bHLH transcription factor, confers water stress resistance by regulating stomatal closure and ROS homeostasis in transgenic tobacco. Front. Plant Sci..

[88] Li B., Gao K., Ren H., Tang W. (2018). Molecular mechanisms governing plant responses to high temperatures. J. Integr. Plant Biol..

[89] Gao X., Cox K.L., He P. (2014). Functions of calcium-dependent protein kinases in plant innate immunity. Plants.

[90] Marcec M.J., Gilroy S., Poovaiah B.W., Tanaka K. (2019). Mutual interplay of Ca(2+) and ROS signaling in plant immune response. Plant Sci..

[91] Andrews S. (2010). https://www.bioinformatics.babraham.ac.uk/projects/fastqc.

[92] Kim D., Paggi J.M., Park C., Bennett C., Salzberg S.L. (2019). Graph-based genome alignment and genotyping with HISAT2 and HISAT-genotype. Nat. Biotechnol..

[93] Liao Y., Smyth G.K., Shi W. (2014). featureCounts: an efficient general purpose program for assigning sequence reads to genomic features. Bioinformatics.

[94] Love M.I., Huber W., Anders S. (2014). Moderated estimation of fold change and dispersion for RNA-seq data with DESeq2. Genome Biol..

[95] Doyle M. (2023).

[96] Shamimuzzaman M., Gardiner J.M., Walsh A.T., Triant D.A., Le Tourneau J.J., Tayal A., Unni D.R., Nguyen H.N., Portwood J.L., Cannon E.K.S. (2020). MaizeMine: A Data Mining Warehouse for the Maize Genetics and Genomics Database. Front. Plant Sci..

