# Supplementary material for: Heat-stress-induced ROS in maize silks cause late pollen tube growth arrest and sterility
Source: iScience. 2024 May 22;27(7):110081. doi: 10.1016/j.isci.2024.110081 (PMC11228802; doi:10.1016/j.isci.2024.110081)
Supplement: Document S1. Figures S1–S9 and Tables S1–S9 [file mmc1.pdf]

iScience, Volume *27*

Supplemental information

Heat-stress-induced ROS in maize silks cause late pollen tube growth arrest and sterility

Wen Gong, Mhaned Oubounyt, Jan Baumbach, and Thomas Dresselhaus

SUPPLEMENTAL MATERIAL

Heat stress induced reactive oxygen species in maize silks cause late pollen tube growth arrest and sterility

Wen Gong, Mhaned Oubounyt, Jan Baumbach, and Thomas Dresselhaus

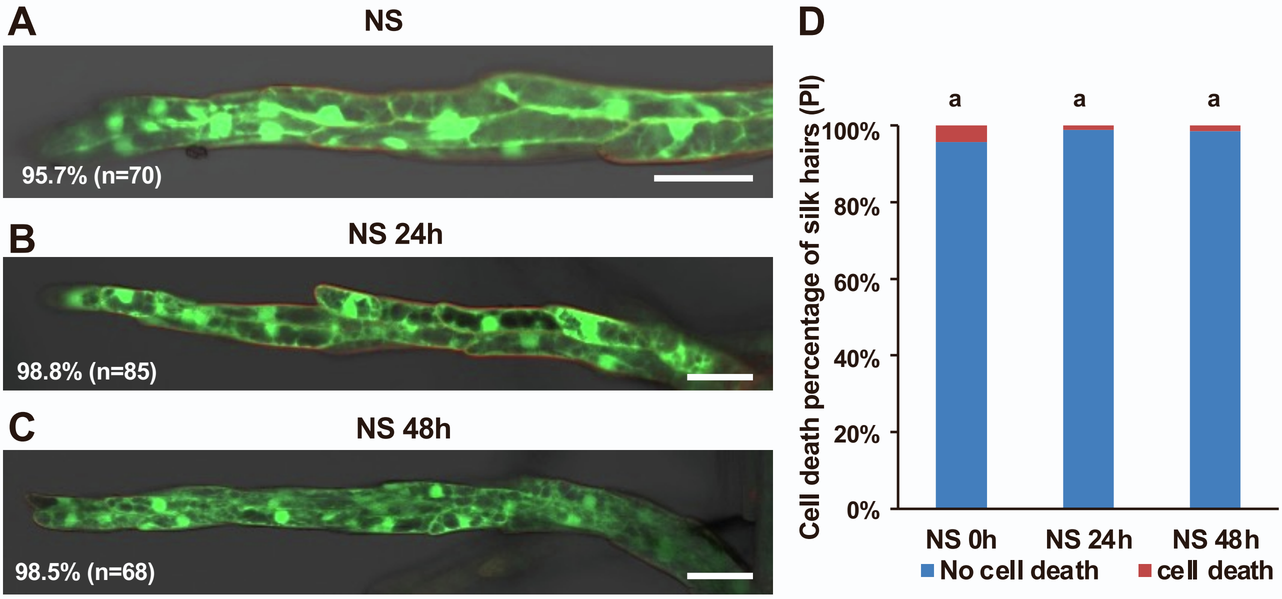

Figure S1. Cell death didn't occur within observed time periods under control conditions, related to Figure 1.

(A-C) Confocal microscopy of maize silk hairs stained with fluorescein diacetate (FDA) and counter stained with propidium iodide (PI). Maize silks (3 days after silk emergence) at control conditions (NS) (A), after 24 hours (B) and 48 hours (C) without heat stress (HS). Percentages are given for silk hairs lacking dead cells. Scale bars: 50 µm. DIC, FDA and PI channels were merged.

(D) Percentage of silk hairs showing cell death (PI-stained nuclei). Letters on columns indicate significantly associated categories. P > 0.05.

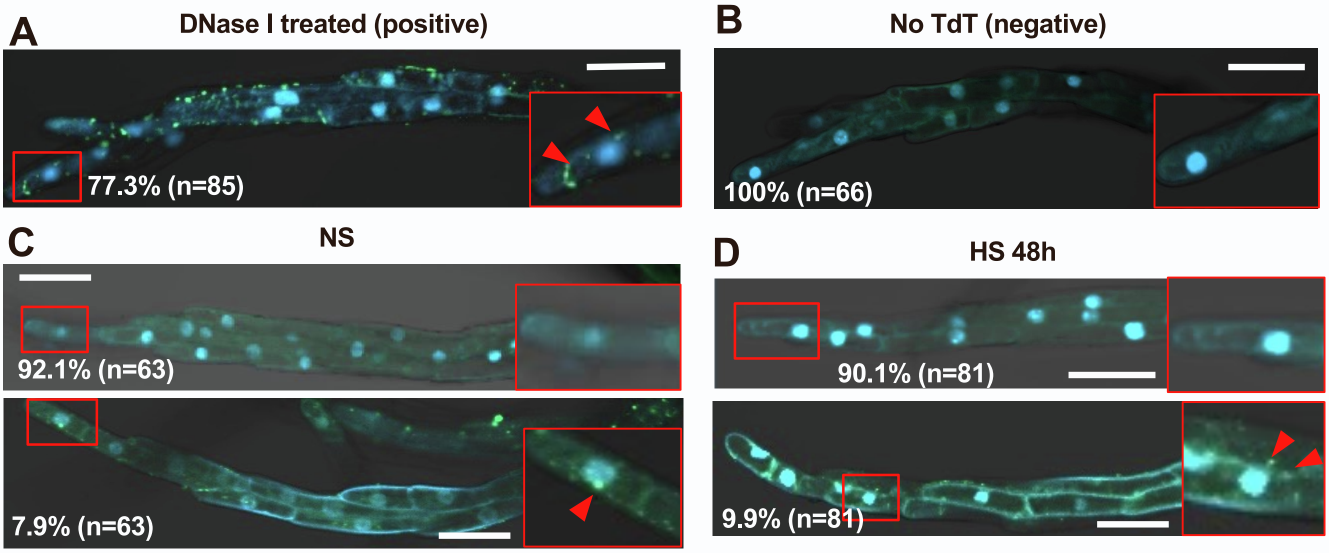

Figure S2. DNA fragmentation appears not to be the prerequisite for heat stress-triggered cell death in maize silk hairs, related to Figure 1.

Confocal microscopy of maize silk hairs after terminal deoxynucleotidyl transferase (TdT) dUTP nick end labeling (TUNEL) assay and counter-stained with 4′,6-diamidino-2-phenylindole (DAPI).

(A) Silks (3 days after emergence) were treated with DNase I before the TUNEL assay and used as a positive control.

(B) Silks without TdT treatment served as a negative control.

(C) Silk hairs (3 days after silk emergence) before heat stress (HS).

(D) Silk hairs (3 days after silk emergence) after 48 HS. Scale bars: 50 μm. Red rectangulars indicate enlarged areas. Red arrowheads indicate signals of fragmented DNA.

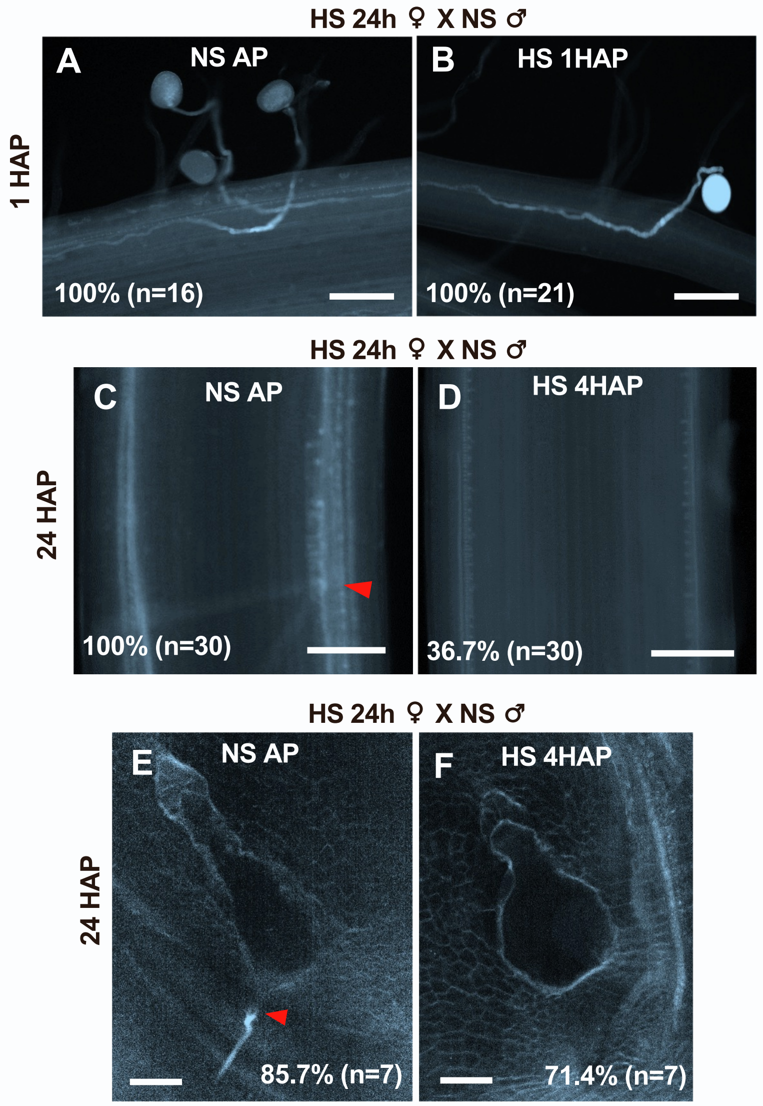

Figure S3. Maize silks exposed to heat stress show inhibition of late pollen tube growth, related to Figure 3.

(A-B) Fluorescent microscopy of aniline blue stained silks 1 hour after pollination (1 HAP), silks (3 days after emergence) after 24 hours HS treatment (HS 24h) were pollinated with NS pollen, then kept in NS condition after pollination for 1 hour (NS AP) (A) or HS exposure for another 1 hours after pollination (HS 1h AP) (B). Scale bars: 100 µm.

(C-D) Fluorescent microscopy of aniline blue stained silks at the 1 to 2 cm proximal region 24 hours after pollination (24 HAP). Silks (3 days after emergence) after 24 hours HS (HS 24h) were pollinated, then kept at NS conditions after pollination (NS AP) (C), or HS exposure for another 4 hours after pollination (D). Red arrowhead indicates a pollen tube in the transmitting tract of silks. Scale bars: 100 µm.

(E-F) Fluorescent microscopy of aniline blue staining of ovule section which contains the female gametophyte and the micropylar region at 24 HAP. Silks (3 days after emergence) after 24 hours HS (HS 24h) were pollinated, then kept at NS conditions after pollination (NS AP) (E) or exposed for another 4h HS after pollination (F). Red arrowhead indicates ae pollen tube at the micropylar region. Scale bars: 50 µm.

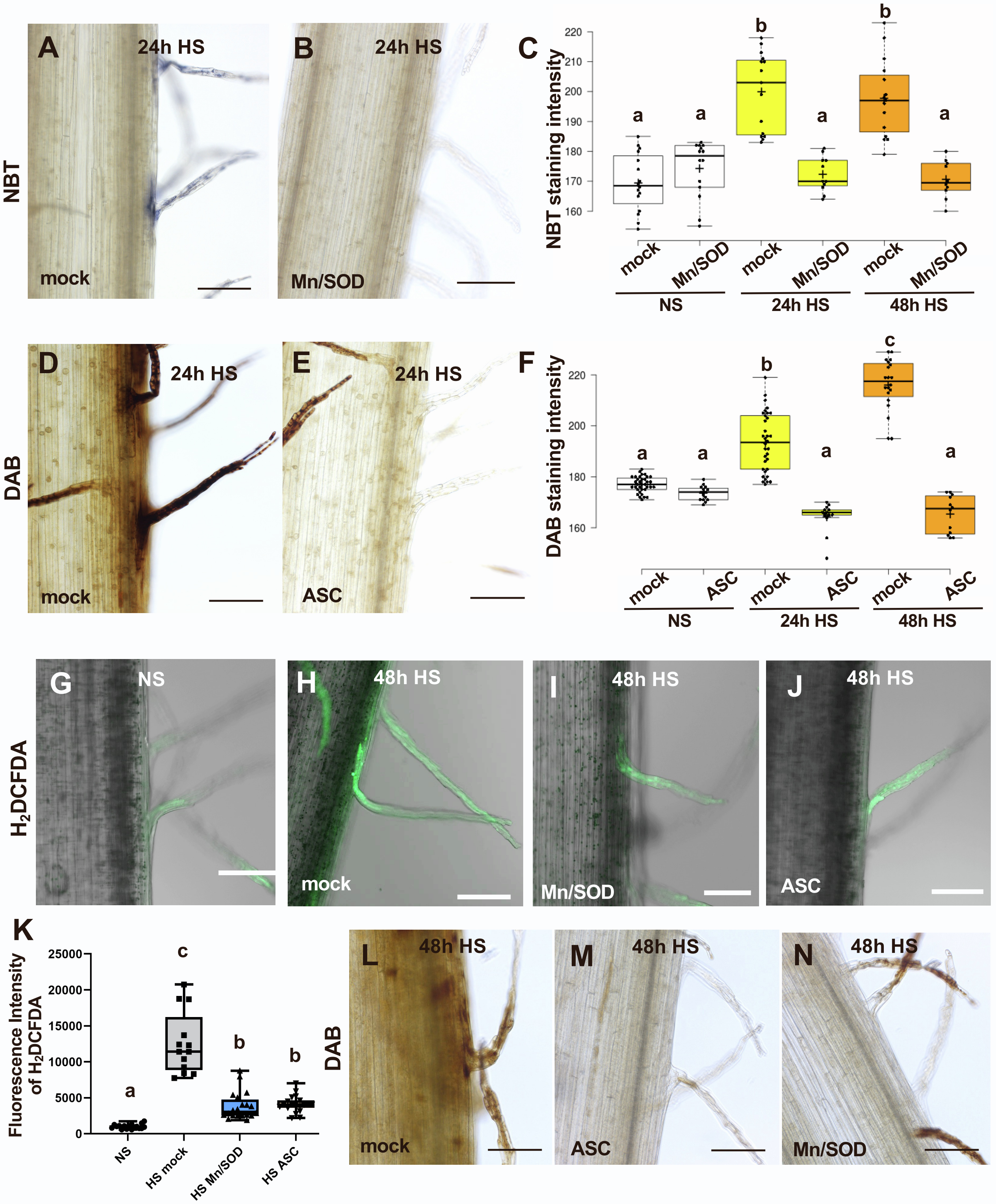

Figure S4. ROS levels are increased in silks under heat stress and ROS scavengers reduce their levels, related to Figure 4.

(A-B) DIC microscopy of nitroblue tetrazolium (NBT) stained silks sprayed with mock solution (A) or sprayed with combined $MnCl_2$ and SOD solution (B). Silks (3 days after emergence) were exposed to HS for 24 hours (HS 24h). (C) Quantification of mean grey values of NBT stained silks. Data includes 3 biological replicates. Letters indicate significance categories. $P < 0.01$ by ANOVA test. (D-E) DIC microscopy of 3,3′-diaminobenzidine (DAB) stained silks sprayed with mock solution (A) or sprayed with $MnCl_2$/SOD solution (B). Silks (3 days after emergence) were exposed to HS for 24 hours (HS 24h). Scale bars: 100 µm. (F) Quantification of mean grey values of DAB stained silks. Data includes 3 biological replicates. Letters indicate significance categories. $P < 0.01$ by ANOVA test. (K) Quantification of relative fluorescence intensity of silks with a H2DCFDA probe. Letters indicate significance categories. $P < 0.01$ by one-way ANOVA-TUKEY test. (L-N) DIC microscopy of DAB stained silks sprayed with mock solution (L) or sprayed with ascorbic acid (ASC) solution (M) or $MnCl_2$/SOD solution (N) during 48h HS treatment. Scale bars: 100 µm.

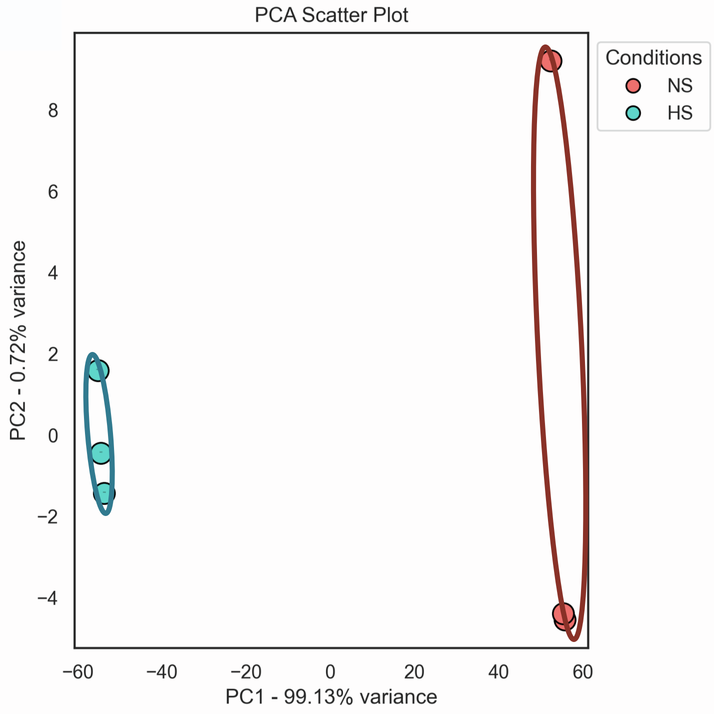

Figure S5. Principal component analysis (PCA) of RNA-seq samples from maize silks, related to Figure 6.

Principal component analysis (PCA) indicating the expression correlation among silks samples at HS and NS conditions. Principal component 1 (PC1) and PC2 separate samples by 99% and 1%, respectively. Circles mark highly correlated samples.

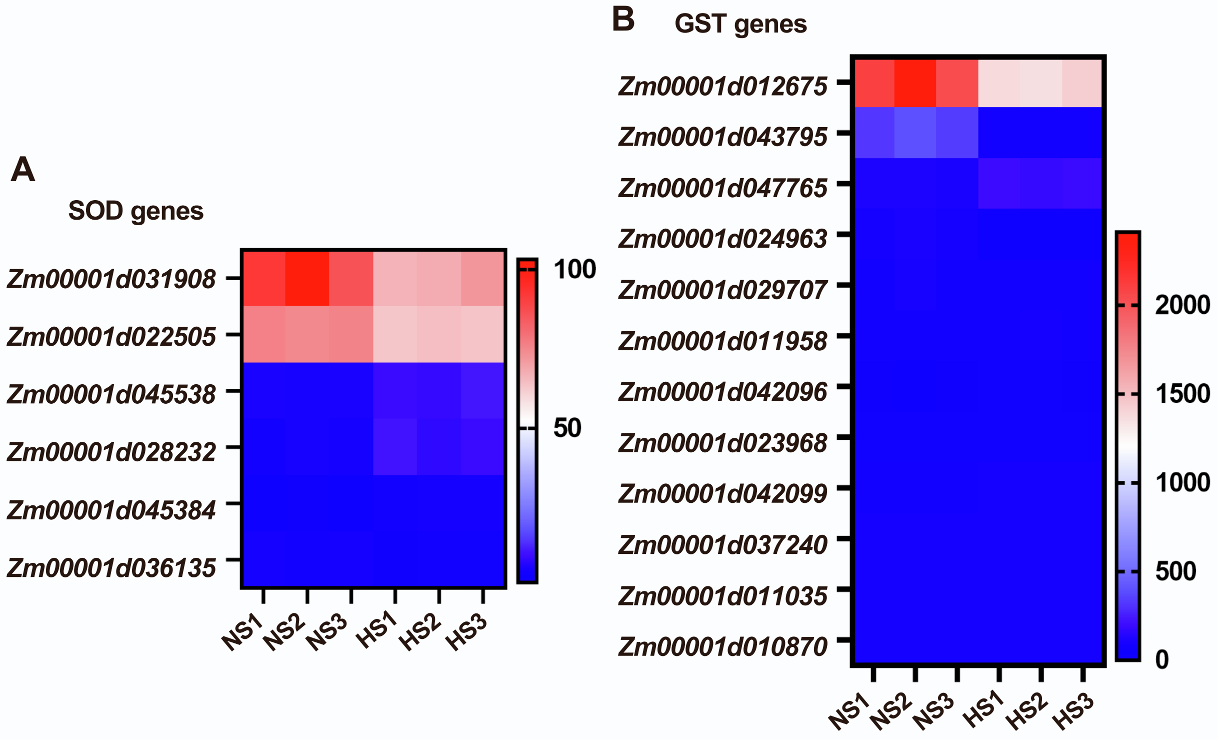

Figure S6. Expression levels of ROS-related gene categories in maize silks after heat stress exposure, related to Figure 6.

(A) Comparable heatmap of gene expression levels of superoxide dismutase (SOD) genes. The numbers on the color scale indicate the TPM values.

(B) Comparable heatmap of gene expression levels of glutathione S-transferase (GST) genes. The numbers on the color scale indicate the TPM values.

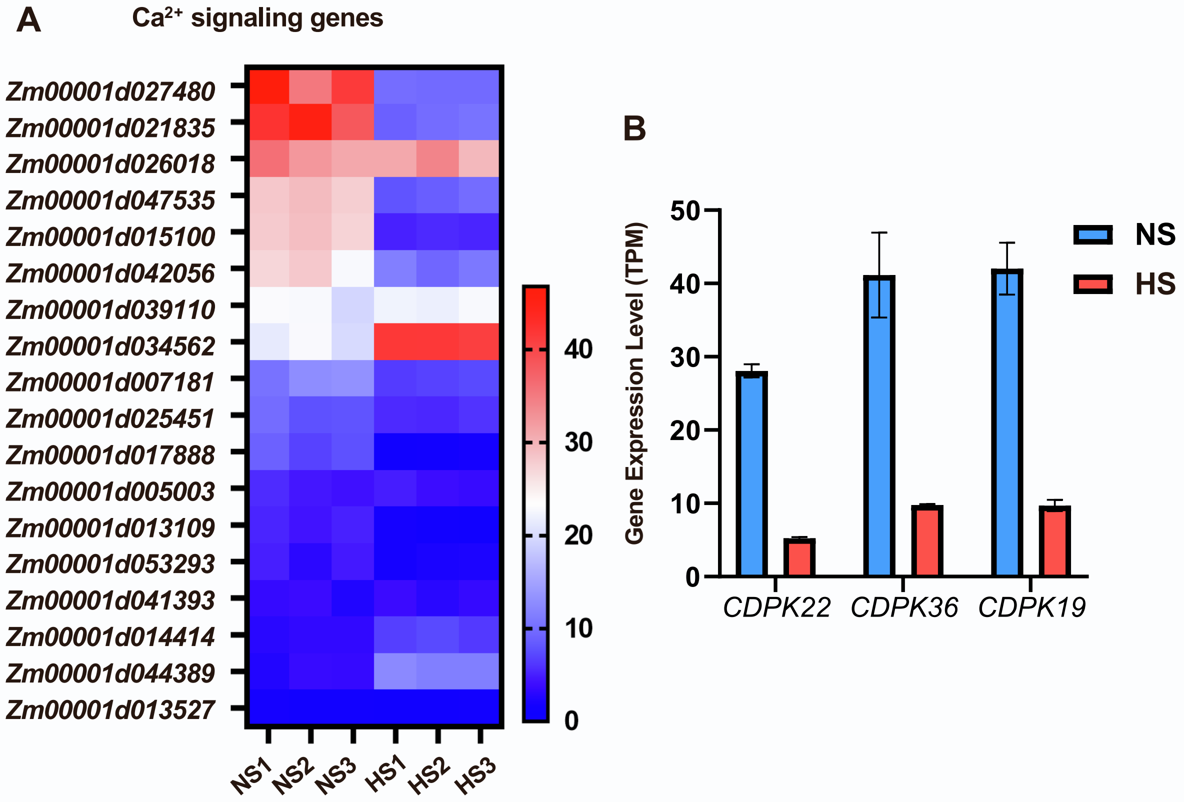

Figure S7. Expression levels of genes in heat stress exposed maize silks involved in Ca^{2+} signaling, related to Figure 6.

(A) Heatmap of the gene expression level of calcium signaling. The numbers on the color scale indicate the TPM values.

(B) The gene expression level (TPM) of top 3 most differentially expressed CDPK genes.

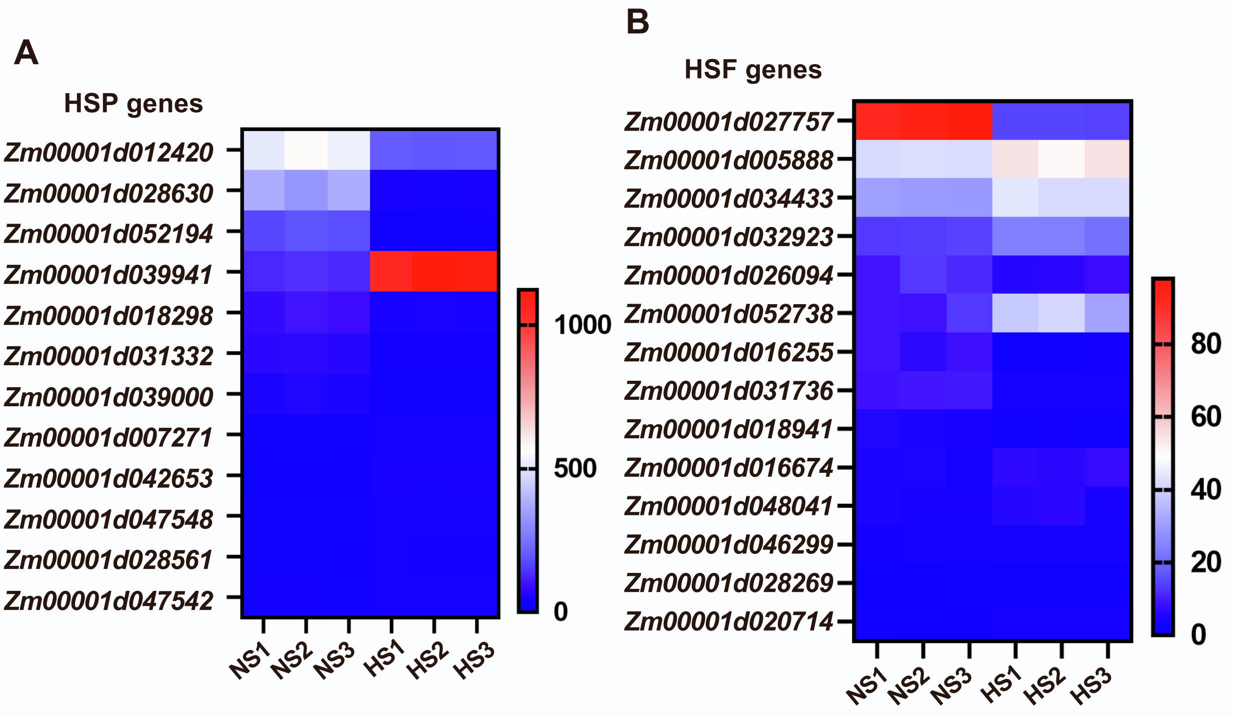

Figure S8. Expression level of genes involved in heat stress responses, related to Figure 6.

(A) Heatmap showing the expression level of maize genes encoding heat shock proteins (HSP). The numbers on the color scale indicate the TPM values.

(B) Heatmap showing the expression level of genes encoding heat shock factor (HSF) transcription factors. The numbers on the color scale indicate the TPM values.

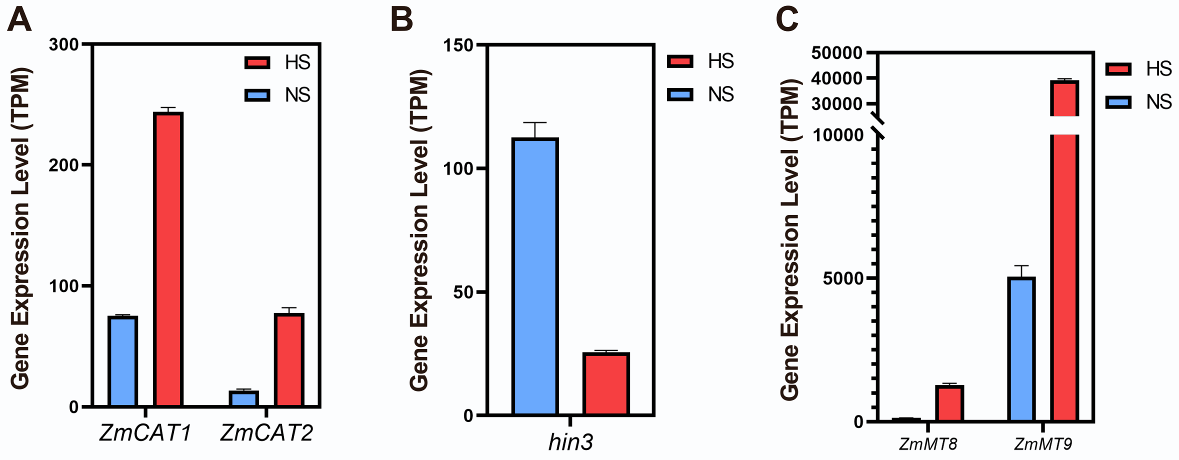

Figure S9. Expression level of genes involved in ROS regulation, related to Figure 6.

(A) Gene expression levels (TPM) of differentially expressed catalase (CAT) genes.

(B) Gene expression level (TPM) of *hin3*, a homologous gene of *botryoid pollen* 1 (*bp*1) in maize.

(C) Gene expression level (TPM) of differentially expressed metallothionein (MT) genes.

Table S1. RNA-seq samples and numbers of genes detected, related to Figure 6.

Sample No.	Sample Content	Reads	Number of genes (TPM>1)
1	NS 1	22,691,412	25,371
2	NS 2	25,060,234	25,356
3	NS 3	22,589,878	25,238
4	HS 1	24,608,961	24,501
5	HS 2	22,812,146	24,283
6	HS 3	21,432,868	24,260

Table S2. Log2FC values of peroxidase (POD) genes, related to Figure 6. Gene identifier (Gene ID) and BaseMean (average of normalized count values divided by size factors taken over all samples) values are provided. Log2FC indicates transcript's expression level changed between HS and NS groups. P-value indicates test for transcripts between HS and NS groups and padj the adjusted P-value for multiple testing of transcripts.

Gene ID	baseMean	log2FC	P-value	padj
Zm00001d031635	833.6883	-1.50482	4.82E-51	6.51E-50
Zm00001d027411	3713.848	-1.5191	1.76E-98	4.39E-97
Zm00001d029747	1402.312	-1.53084	4.99E-77	9.8E-76
Zm00001d010924	3393.763	-1.78706	1.9E-113	5.5E-112
Zm00001d037061	750.2089	-2.09762	1.64E-72	3.04E-71
Zm00001d038599	109.3555	-2.23245	8.51E-14	3.99E-13
Zm00001d022453	111.1806	-2.67078	1.79E-22	1.23E-21
Zm00001d018619	402.387	-2.80226	4.8E-56	7.01E-55
Zm00001d034129	1381.328	-2.84535	1.3E-164	5.9E-163
Zm00001d002899	16607.04	-3.03986	0	0
Zm00001d023899	291.9643	-3.1544	1.47E-55	2.14E-54
Zm00001d042022	12441.31	-3.21729	0	0
Zm00001d016182	411.2284	-3.21972	6.73E-89	1.51E-87
Zm00001d014467	882.0996	-3.47201	8.9E-146	3.6E-144
Zm00001d040399	1061.855	-3.73129	1.8E-264	1.5E-262
Zm00001d052335	3.079455	-4.08082	0.025201	0.041604
Zm00001d002898	50.20101	-4.21998	1.94E-16	1.04E-15
Zm00001d014608	950.0055	-4.71711	2.8E-271	2.5E-269
Zm00001d008266	124.4604	-4.96299	1.38E-40	1.49E-39
Zm00001d053554	15.66823	-5.5821	2.93E-06	8.19E-06
Zm00001d043238	445.4333	-5.7096	5.4E-127	1.8E-125
Zm00001d016185	7.288543	-6.3616	3.51E-05	8.77E-05
Zm00001d008173	56.00531	-6.43972	5.21E-16	2.75E-15
Zm00001d046184	233.8563	-6.49398	3.59E-58	5.4E-57
Zm00001d040581	82.95971	-9.872	3.56E-16	1.89E-15
Zm00001d046186	85.88679	-9.92231	2.04E-16	1.09E-15

Table S3. Top 50 DEGs from enriched TF families, related to Figure 7. Gene identifier (Gene ID), TF family as well as BaseMean (average of normalized count values divided by size factors taken over all samples) values are provided. Log2FC indicates transcript's expression level changed between HS and NS groups. P-value indicates test for transcripts between HS and NS groups and padj the adjusted P-value for multiple testing of transcripts.

Gene ID	Family	baseMean	log2FC	P-value	padj
Zm00001d043706	bHLH	304.2032	-11.7465	4.77E-23	3.35E-22
Zm00001d024522	bHLH	3567.405	-9.91921	1.7E-228	1.2E-226
Zm00001d051149	MYB	78.57449	-9.79331	1.02E-15	5.31E-15
Zm00001d025141	bHLH	77.5669	-9.77585	8.05E-16	4.2E-15
Zm00001d014989	MYB	76.67973	-9.75901	7.92E-16	4.14E-15
Zm00001d012642	bHLH	71.05397	-9.64868	2.27E-15	1.16E-14
Zm00001d052397	MYB	46.58341	-9.03952	2.06E-13	9.46E-13
Zm00001d028842	MYB	44.6873	-8.97962	3.3E-13	1.5E-12
Zm00001d012482	WRKY	38.15826	-8.75184	1.93E-12	8.43E-12
Zm00001d037528	bHLH	33.75785	-8.57565	7.92E-12	3.35E-11
Zm00001d050247	WRKY	33.38392	-8.55892	7.81E-12	3.3E-11
Zm00001d034596	bHLH	108.891	-8.40549	1.08E-15	5.62E-15
Zm00001d032265	WRKY	29.32114	-8.3707	5.79E-11	2.32E-10
Zm00001d036118	HD-ZIP	189.6645	-8.21025	3.36E-28	2.73E-27
Zm00001d018119	bHLH	24.63052	-8.12037	1.94E-10	7.53E-10
Zm00001d020540	AP2	47.34161	-8.09679	4.8E-11	1.93E-10
Zm00001d020540	ERF	47.34161	-8.09679	4.8E-11	1.93E-10
Zm00001d045400	HD-ZIP	23.59559	-8.05849	3.5E-10	1.34E-09
Zm00001d037140	HD-ZIP	21.24797	-7.90613	4.53E-09	1.6E-08
Zm00001d041853	MYB	18.60669	-7.71606	4.12E-09	1.46E-08
Zm00001d016457	WRKY	18.51621	-7.70832	3.75E-09	1.34E-08
Zm00001d035266	NAC	16.50903	-7.54233	1.41E-08	4.8E-08
Zm00001d004744	MYB	16.0312	-7.50125	2.04E-08	6.88E-08
Zm00001d004744	MYB_related	16.0312	-7.50125	2.04E-08	6.88E-08
Zm00001d034920	ERF	287.2622	-7.47802	1.26E-54	1.8E-53
Zm00001d036726	WRKY	283.1162	-7.45994	1.23E-53	1.73E-52
Zm00001d009619	WRKY	14.96222	-7.40041	4.13E-08	1.36E-07
Zm00001d041981	ERF	11.77585	-7.05611	3.34E-07	1.02E-06
Zm00001d018225	HD-ZIP	41.15593	-6.99411	1.48E-10	5.78E-10
Zm00001d044272	bHLH	10.75476	-6.92521	6.97E-07	2.07E-06
Zm00001d030069	HD-ZIP	249.4806	-6.78539	1.94E-58	2.95E-57
Zm00001d031796	ERF	9.734158	-6.78131	1.65E-06	4.72E-06
Zm00001d018191	ERF	32.89597	-6.6735	2.44E-09	8.79E-09
Zm00001d045107	bHLH	8.237471	-6.53967	9.39E-06	2.49E-05
Zm00001d038216	ERF	58.29874	-6.48244	3.39E-16	1.81E-15
Zm00001d020705	bHLH	142.5266	-6.45699	4.27E-37	4.32E-36
Zm00001d017366	ERF	15.18466	-6.44287	2.58E-06	7.26E-06
Zm00001d002285	NAC	8.45378	6.484361	7.36E-06	1.97E-05
Zm00001d008399	NAC	16.51552	6.475225	1.41E-06	4.09E-06
Zm00001d044975	MYB	36.80446	5.714799	8.3E-12	3.51E-11
Zm00001d049058	ERF	286.4809	5.552055	7.81E-81	1.59E-79
Zm00001d009622	ERF	3.794951	5.32383	0.002297	0.004533
Zm00001d051509	bHLH	3.56481	5.236061	0.003083	0.00597
Zm00001d021205	ERF	9.46116	4.744487	0.000338	0.000749
Zm00001d045600	NAC	14.33843	4.325735	1.25E-05	3.29E-05
Zm00001d028999	NAC	662.5717	4.010964	4.2E-170	2E-168
Zm00001d022224	bHLH	22.93363	3.980442	2.2E-08	7.39E-08
Zm00001d025823	MYB_related	8.535509	3.978882	0.001373	0.002806
Zm00001d017592	ERF	5.058456	3.793715	0.029596	0.048145
Zm00001d005892	ERF	26.95525	3.614141	3.5E-07	1.07E-06
